# A Key to Material's Stability: Tuning Pyrolysis Temperature in SnS*_x_* @C Anodes for Sodium‐Ion Batteries

**DOI:** 10.1002/smll.202504485

**Published:** 2025-07-31

**Authors:** Zuzanna Zarach, Mirosław Sawczak, Carsten Dosche, Konrad Trzciński, Mariusz Szkoda, Magdalena Graczyk‐Zając, Ralf Riedel, Gunther Wittstock, Andrzej P. Nowak

**Affiliations:** ^1^ Faculty of Chemistry Gdansk University of Technology Gdansk 80‐233 Poland; ^2^ Centre of Plasma and Laser Engineering The Szewalski Institute of Fluid‐Flow Machinery of Polish Academy of Sciences Gdansk 80‐231 Poland; ^3^ Institute of Chemistry Carl von Ossietzky University of Oldenburg 26111 Oldenburg Germany; ^4^ Institute of Materials Science Darmstadt University of Technology 64287 Darmstadt Germany; ^5^ EnBW Energie Baden‐Wurttemberg AG 76131 Karlsruhe Germany

**Keywords:** anode materials, metal chalcogenides, operando Raman spectroscopy, sodium‐ion batteries, tin sulfide

## Abstract

Developing robust and efficient anodes is essential for advancing sodium‐ion battery technology. Herein, a systematic investigation of SnS*_x_* @C composites prepared at different pyrolysis temperatures to elucidate how their structural, surface, and electrochemical properties govern sodium‐ion storage is reported. The study reveals that a lower synthesis temperature traps extra sulfur within the carbon matrix, which hampers the complete SnS conversion reaction and Na^+^ intercalation processes. In contrast, pyrolysis at 800 °C facilitates more thorough sulfur release, yielding a defect‐rich but stable carbon matrix that supports enhanced sodiation/desodiation reversibility. *Operando* Raman spectroscopy and X‐ray photoelectron spectroscopy depth profiling confirm that the pyrolysis temperature strongly affects the formation and stability of the solid electrolyte interphase. The SnS*_x_* @C material pyrolyzed at 800 °C not only possesses superior ion transport characteristics but also delivers enhanced electrochemical performance, maintaining a stable capacity of ≈500 mAh g^−1^ at *C*/10 and retaining a substantial fraction of its capacity over 100 cycles, in contrast to the rapidly decaying capacity of the material pyrolyzed at 600 °C.

## Graphical abstract

This study shows how tuning pyrolysis temperature impacts the structure and performance of SnS*_x_* @C anodes in sodium‐ion batteries. Using *operando* Raman spectroscopy, the authors reveal that treatment at 800 °C enhances sodium storage and long‐term stability. The findings highlight the role of carbon defects and solid electrolyte interphase formation in improving battery performance.

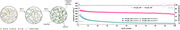

## 1 Introduction

In today's world, the rapidly growing demand for electrical energy and the increasing electrification of various sectors require continuous advancements in energy storage technologies. This demand spans from large‐scale, stationary energy storage systems to portable devices, including electric vehicles (EVs), where the performance of batteries is paramount. The need for longer driving ranges, faster charging, and higher overall energy density in EVs has driven scientific research and innovation in advanced battery systems. While significant progress has been made in lithium‐ion batteries (LIBs) technology over the past decade,^[^ ^1^ ^]^ the limited supply of lithium, along with financial concerns, has led to increased scrutiny of this technology's long‐term viability.^[^ ^2^, ^3^ ^]^

Lithium's scarcity arises from its low elemental abundance in the Earth's crust and the geographically concentrated nature of its reserves. As demand grows, particularly due to the exponential uptake of electric vehicles and portable electronics, the price of lithium has surged, prompting the exploration of alternative energy storage solutions. Sodium‐ion batteries (SIBs) have emerged as a promising contender due to the abundant availability of sodium, which is two to three orders of magnitude more plentiful than lithium, both on land and in the oceans.^[^ ^4^, ^5^ ^]^ Although early research into sodium‐based battery technology dates back to the 1970s, LIBs gained traction due to several key breakthroughs^[^ ^6^, ^7^, ^8^ ^]^ resulting in a slowdown of sodium‐ion research. However, given the increasing disparity between lithium supply and demand, SIBs are now being revisited as a cost‐effective alternative for large‐scale energy storage.^[^ ^9^, ^10^ ^]^ Yet, several challenges are to be faced when SIBs are compared to their lithium‐ion counterparts, particularly in terms of energy density. Sodium ions are larger (1.02 Å vs 0.76 Å for Li^+^) and heavier (23 g mol^−1^ vs 6.9 g mol^−1^ for Li^+^), which impede the achievement of high volumetric and gravimetric energy densities.^[^ ^11^ ^]^ Commercial anode materials like graphite, which are well‐suited for LIBs, do not perform optimally with sodium ions due to significant structural mismatches, resulting in poorer capacity retention during long‐term cycling.^[^ ^12^, ^13^, ^14^ ^]^ Nevertheless, SIBs offer several advantages that make them attractive alternatives. For instance, SIBs utilize aluminum current collectors, which do not form alloys with sodium, improving their safety profile compared to LIBs. Moreover, the slightly smaller Stokes radius of sodium ions (e.g., in propylene carbonate (PC): Na^+^ (4.6 Å) < Li^+^ (4.8 Å)) improves their diffusion kinetics at the solid‐electrolyte interphase (SEI), leading to enhanced ionic conductivity.^[^ ^15^ ^]^ This can help mitigate some of the performance drawbacks associated with the larger ionic size and mass of sodium, offering potential for further improvements in energy efficiency and cycle life.^[^ ^16^ ^]^ Furthermore, many SIBs designs employ stable insertion‐based or carbon‐based anodes that limit dendritic plating, thereby enhancing their inherent safety and suitability for large‐scale applications.^[^ ^17^, ^18^ ^]^ However, dendrite formation remains inevitable when metallic sodium is used directly as the anode material, and thus, the search for suitable anode materials continues to be a key challenge in improving the performance and safety of SIBs.

Within the spectrum of emerging anodes, metal sulfides (MeS*_x_*) have attracted attention due to their favorable electrochemical properties relative to their oxide counterparts. The inherent features of MeS*_x_*, including tunable crystal structures, layered architectures, and flexible valence states, contribute to their higher electronic conductivity and relatively faster sodium storage kinetics in comparison with the corresponding oxides.^[^ ^19^, ^20^, ^21^ ^]^ This faster kinetic profile is attributed to the smaller Me─S bond energy in MeS*_x_*, which facilitates a swifter conversion reaction than is possible with Me─O bonds in metal oxides.^[^ ^22^, ^23^ ^]^ As such, many metal sulfides, including those based on Mo, W, Fe, and Sn, exhibit multiple reaction mechanisms for sodium storage, including intercalation, conversion and alloying reactions, which together support enhanced charge capacity and extended cycle life. Within the MeS*_x_* group, tin‐based sulfides stand out due to their exceptional theoretical capacities. Tin(II) sulfide, for instance, exhibits a high theoretical capacity of 1022 mAh g^−1^, resulting from a sequence of sodium storage reactions that involve initial Na^+^ ion intercalation, followed by conversion to Na_2_S and eventual alloying with Na to produce Na_3.75_Sn.^[^ ^24^ ^]^ These multiple reaction pathways contribute to its impressive energy density but also lead to notable challenges, primarily associated with structural integrity over repeated cycles. During the sodiation/desodiation process, SnS may experience substantial volume changes as bonds between Sn and S are repeatedly formed and broken. This causes electrode expansion, pulverization, and degradation, which can result in reduced cycling stability.^[^ ^25^ ^]^

A key determinant of battery performance and lifespan is the formation and stability of an SEI. In carbon‐based anodes, such as hard carbon, the SEI is governed by the material's porosity, surface functionalities, and defect structures, which can store sodium via multiple mechanisms—ranging from surface adsorption at defect sites to intercalation between the domains and pore filling at low voltages^[^ ^26^, ^27^, ^28^, ^29^ ^]^—making the SEI structure equally complex and dynamic. On the other hand, metal sulfide anodes undergo repeated conversion and alloying reactions that continuously reshape the electrode–electrolyte interface. This restructuring, with repeated structural expansion and contraction, can fracture the SEI, triggering persistent electrolyte decomposition, an increase in charge transfer resistance, and subsequent capacity fading.^[^ ^21^, ^25^, ^30^ ^]^ Recent studies have highlighted the importance of designing anodes with controlled porosity and tailored surface chemistry to promote the formation of an inorganic‐rich, uniform, and stable SEI, which can mitigate these effects.^[^ ^29^, ^31^, ^32^ ^]^ Especially, SEI layers with a well‐structured inorganic composition—such as NaF, Na_2_O, or Na_2_S—have demonstrated improved stability and better Na^+^ transport kinetics, effectively reducing side reactions and enhancing long‐term performance.^[^ ^33^ ^]^ Thus, designing SnS‐based composite anodes in which carbon phases mitigate volume changes, while promoting stable SEI formation, is one of the most viable approaches to achieving better cycling longevity and rate capability.

Understanding and controlling the SEI formation, as well as degradation mechanisms responsible for capacity loss, is therefore essential to advancing the stability of SIBs. Advanced in situ and *operando* techniques, including Raman spectroscopy, have proven invaluable for real‐time monitoring of the dynamic processes occurring during charge–discharge cycles. These methods provide crucial insights into the formation stability of the SEI, but also for optimizing material design and electrolyte composition.^[^ ^34^, ^35^ ^]^ Gan et al.^[^ ^36^ ^]^ employed *operando* Raman spectroscopy to investigate a nitrogen‐doped hard carbon anode for SIBs, revealing the presence of C─C• and C─N• radicals that significantly contributed to additional sodium storage sites. Raman measurements enabled the identification of reversible radical evolution during sodiation and desodiation, which was directly linked to the enhanced capacity and long‐term cycling stability.^[^ ^36^ ^]^ Similarly, Weaving et al.^[^ ^37^ ^]^ used *operando* Raman spectroscopy to elucidate the sodiation mechanism in commercial hard carbon and demonstrated how changes in the *I*_G_ and *I*_D1_ Raman bands corresponded to sodium intercalation between turbostratic nanodomains (TNDs). The findings showed that sodium occupation of edge sites and interlayer defects drives the sloping voltage profile, while pore filling dominates at low potentials. Importantly, the study emphasized the necessity of operando techniques, as ex situ measurements often fail to capture critical transient states, such as those associated with SEI formation or sodium reversibility.^[^ ^37^ ^]^

Thus, in this work, SnS*_x_* @C composites for sodium‐ion battery anode material are investigated, using *operando* Raman spectroscopy, thereby unveiling the differences that emerge during intercalation processes within the carbon material. The study also explores the impact of pyrolysis temperature on the material's surface properties, porosity, and structural features, which collectively influence the efficiency of sodium‐ion storage and stability of chalcogenide‐based anode materials. Furthermore, X‐ray photoelectron spectroscopy depth profiling is employed to elucidate how synthesis protocols and thermal treatments affect the electrochemical performance. Overall, the findings provide critical insights into tuning the SnS*_x_* @C electrode architectures for high‐performance sodium‐ion anodes and emphasize the role of advanced spectroscopic techniques in guiding the design of energy storage materials.

## 2 Results and Discussion

**Figure 1:**
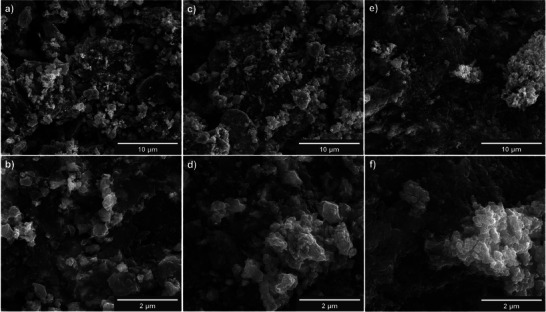
Scanning electron microscopy images of a,b) SnS*_x_* @C_600, c,d) SnS*_x_* @C_800, and e,f) SnS*_x_* @C_1000 materials.

Regardless of the temperature applied during the annealing process, the SEM images reveal a distinct irregularity within the morphology, accompanied by a similarity in particle sizes (**Figure** **1**). In addition, the observations indicate a tendency of the materials to form agglomerates, suggesting that the thermal treatment influences the degree of particle clustering. However, the measurements conducted with the energy‐dispersive X‐ray spectroscopy (EDX) show a change in stoichiometry of the tin‐sulfide compound (Tables S1–S4, Supporting Information). The sulfur‐to‐tin ratio changed from 2:1 for the untreated sample (SnS*_x_* @C), to a 1:0.92 ratio for SnS*_x_* @C_600 and a 1:0.95 ratio for SnS*_x_* @C_800. Also, a significant loss of both sulfur and tin is found for SnS*_x_* @C_1000, indicating the upper practical limit for the thermal stability of tin‐sulfide compounds.

**Figure 2:**
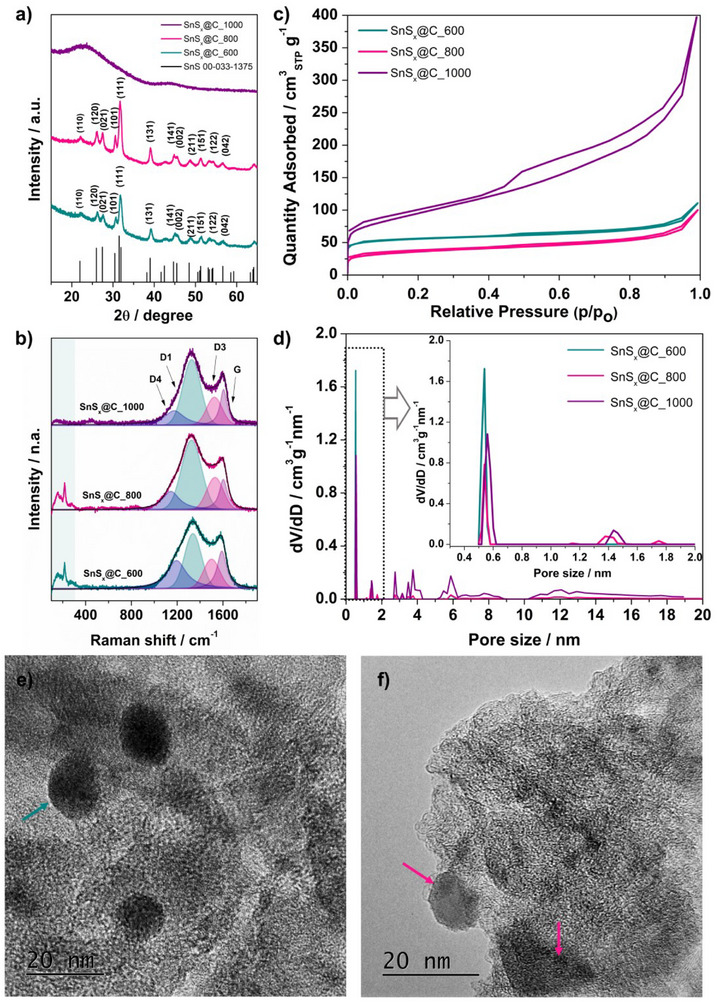
a) XRD patterns, b) Raman spectra, c) nitrogen adsorption–desorption isotherms, and d) pore size distribution calculated from N_2_ adsorption isotherms based on the DFT method for SnS*_x_* @C materials; TEM images for e) SnS*_x_* @C_600 and f) SnS*_x_* @C_800.

The EDX results, confirming the presence of Sn and S atoms at different stoichiometries, were consistent with the X‐ray diffraction (XRD) data—the obtained patterns are presented in **Figure** **2**a. The hydrothermal synthesis resulted in the synthesis of the pure, single‐phase SnS_2_ (see Figure S2, Supporting Information, ICDD‐PDF 00‐001‐1010). This is confirmed by the diffraction peaks registered at 28.7°, 32.9°, 50.3°, and 52.9° that correspond to the (100), (101), (110), and (111) planes of the hexagonal SnS_2_, respectively.^[^ ^38^ ^]^ Since it is impossible to distinguish the reflection from the (001) plane at 15.0°, it is likely that the adsorption of carbon species on this plane suppresses its detection in the XRD pattern.^[^ ^39^ ^]^

The thermal treatment of the SnS_2_@C material in Ar atmosphere at elevated temperatures reaching 800 °C led to the conversion of tin(IV) sulfide to tin(II) sulfide. For both SnS*_x_* @C_600 and SnS*_x_* @C_800, the XRD patterns indicate the formation of orthorhombic SnS (ICDD‐PDF 00‐033‐1375).^[^ ^40^ ^]^ No reflections attributable to SnS_2_, Sn_2_S_3_, or metallic Sn are present after pyrolysis. However, the treatment at 1000 °C resulted in losing the chalcogenide‐based part of the composite with the increased contribution of an amorphous‐type carbon structure, since the pattern shows only the broad carbon (002) reflex at 24°. On the basis of the obtained results, the crystallite size was calculated by utilization of Scherrer's equation:

(1) $$D=\frac{K\lambda }{\beta \cos\theta }\;$$

where *D* represents the crystallite size, *K* is a constant (0.94), *λ* is the X‐ray wavelength, *β* is the width at half maximum of the peak, and *θ* is the Bragg angle. The (111) reflection of SnS at 31.6° narrows from 0.78 to 0.72 (FWHM) when the pyrolysis temperature is raised from 600 to 800 °C, corresponding Scherrer sizes of 110.3 and 120.3 Å, respectively. High‐resolution transmission electron microscopy (TEM) supports these X‐ray calculations. In the SnS*_x_* @C‐600 sample, individual SnS particles are visible in the carbon matrix (Figure 2e). The small difference in size is expected since TEM images include a thin carbon shell and can show two overlapping crystals as one. Moreover, at 800 °C, SnS is still present as nanodomains (Figure 2f and Figure S3a, Supporting Information), but fewer particles are seen: at this temperature, tin and sulfur start to evaporate, and neighboring grains start to sinter, so their edges are less distinct. In the SnS*_x_* @C_1000, no SnS particles are detected—TEM shows only the porous carbon framework (Figure S3b, Supporting Information), which is consistent with XRD results. Also, some differences were revealed by Raman spectroscopy analysis, the results of which are presented in Figure 2b (with a narrow range shown in Figure S4a, Supporting Information), revealing four main carbon‐related bands—D1, D3, D4, and the G band—across all samples.^[^ ^41^ ^]^ All spectral parameters obtained from the deconvoluted Raman spectra are listed in Table S5 (Supporting Information). The G band (1590—1605 cm^−1^) is typically assigned to the in‐plane stretching between sp^2^ carbon atoms, whereas the D1 band (1324–1337 cm^−1^) arises from disordered, defect‐activated modes and the vibration of graphene layer edges.^[^ ^27^ ^]^ Two additional disorder‐related features, D3 (1500–1525 cm^−1^) and D4 (1170–1195 cm^−1^), were also deconvoluted. For the SnS*_x_* @C_1000 sample, only these four carbon bands are visible, indicating the almost complete loss of chalcogenide‐based phases at this temperature, which is in agreement with the XRD data.

In contrast, both SnS*_x_* @C_600 and SnS*_x_* @C_800 show additional bands at ≈158.7, 181.2, and 217.7 cm^−1^_,_ corresponding to the B_3g_, B_2g_, and A_g_ vibrational modes of SnS, respectively.^[^ ^42^ ^]^ Notably, the intensities of these SnS bands can vary substantially with the laser excitation wavelength owing to resonance effects in chalcogenide‐based materials.^[^ ^43^ ^]^ Here, an excitation of 785 nm (≈1.6 eV) closely matches the direct bandgap of SnS (≈1.5 eV), enhancing those vibrational features.^[^ ^44^ ^]^ Despite the usual expectation that higher‐temperature annealing produces more ordered carbon (i.e., lower *I*_D1_/*I*_G_ ratio), the *I*_D1_/*I*_G_ ratio (see Table S5, Supporting Information) actually increases from 1.44 for SnS*_x_* @C_600 to 2.07 for SnS*_x_* @C_800, before dropping slightly to 1.84 for SnS*_x_* @C_1000. Although it is not a common phenomenon, a similar trend was also reported by Li et al.^[^ ^45^ ^]^ and Simone et al.^[^ ^32^ ^]^ for hard carbons with SIBs application. The higher *I*_D1_/*I*_G_ ratio at 800 °C suggests that sulfur released during pyrolysis may disrupt the carbon structure, generating additional defects. In contrast, at 600 °C, more sulfur appears to remain within the matrix, reducing new defect formation and thus yielding a lower *I*_D1_/*I*_G_ ratio. By the time the temperature reaches 1000 °C, most sulfur‐containing species have sublimated, leaving predominantly carbon (the *I*_D1_/*I*_G_ ratio of 1.84). The moderate decrease in the *I*_D1_/*I*_G_ ratio compared to SnS*_x_* @C_800 suggests a partial reorganization of the carbon framework, likely driven by the high‐temperature annealing process. At the same time, the G‐band width narrows steadily, from 50 cm^−1^ for SnS*_x_* @C_600, 45 cm^−1^ for SnS*_x_* @C_800 to 37 cm^−1^ for SnS*_x_* @C_1000 (Table S5, Supporting Information). According to the Ferrari–Robertson amorphization model, a narrower G band signals larger and more ordered sp^2^ domains.^[^ ^46^, ^47^ ^]^ Thus, the observed values confirm that the overall graphitization degree still increases with temperature even though the *I*_D1_/*I*_G_ ratio is highest for SnS*_x_* @C_800. The blue shift of the G band towards the graphite value (1600 cm^−1^) in the SnS*_x_* @C_1000 further supports this conclusion. Nonetheless, the removal of sulfur at elevated temperatures can also impact the overall electrochemical properties by altering the number and nature of carbon defects available for sodium storage.

Further investigation included N_2_ adsorption measurements, for which the adsorption–desorption isotherms were obtained and are presented in Figure 2c. The isotherms for the SnS*_x_* @C_600 and SnS*_x_* @C_800 exhibit a type‐I behavior, according to IUPAC classification, indicative of a predominantly microporous structure. When the pyrolysis temperature is increased to 1000 °C, a type‐IV isotherm is recorded with a pronounced hysteresis (see SnS*_x_* @C_1000), characteristic of micro‐ and mesoporous carbons. The pore size distribution in Figure 2d indicates that at 800 and 1000 °C, the pore size is in the range of ≈1 to ≈4 nm (see also Figure S4b, Supporting Information), confirming the formation of micro‐mesoporous structure, whereas the SnS*_x_* @C_600 sample remains primarily microporous (pores around 0.5 nm). Interestingly, both the total pore volume (*V*_t_) and surface area (*S*_BET_) in **Table** **1** decrease when a substantial SnS fraction is present. This effect is most pronounced for SnS*_x_* @C_800, which shows *V*_t_ = 0.15 cm^3^ g^−1^, compared to 0.17 cm^3^ g^−1^ for SnS*_x_* @C_600. A likely explanation is that partial sintering and more thorough decomposition of SnS_2_ to SnS cause pore collapse and a reduced overall pore volume at 800 °C. By contrast, pyrolysis to 1000 °C (where tin sulfide largely decomposes and evaporates) leads to a predominantly carbon‐based structure with significantly higher pore volume of 0.62 cm^3^ g^−1^ and a broader size distribution of mesopores.

**Table 1:** Specific surface area calculated with BET theory, total pore volume determined with the DFT method, and crystallite size obtained from the Scherrer equation for SnS*_x_* @C materials.

Sample	*S*_BET_ [m^2^ g^−1^]	*V*_t_ [cm^3^ g^−1^]
SnS*_x_* @C_600	212	0.17
SnS*_x_* @C_800	128	0.15
SnS*_x_* @C_1000	321	0.62

**Figure 3:**
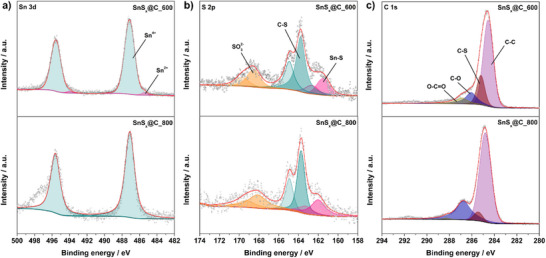
XPS spectra of a) Sn 3d, b) S 2p, and c) C 1s regions for the SnS*_x_* @C_600 and SnS*_x_* @C_800 electrodes.

To further compare the SnS*_x_* @C_600 and SnS*_x_* @C_800 electrode materials, an X‐ray photoelectron spectroscopy (XPS) analysis was performed for layered materials, as shown in **Figure** **3**. Table S6 (Supporting Information) summarizes the corresponding relative elemental composition, revealing that the surface of both materials is dominated by carbon, but the Sn content is significantly lower for SnS*_x_* @C_800 (0.64% compared to 1.34% for SnS*_x_* @C_600), indicating that SnS starts to evaporate from the sample surface already at 800 °C. These results are in contrast to the EDX results with higher Sn and S contents, reflecting the surface‐sensitive nature of XPS versus the more bulk‐sensitive EDX. For SnS*_x_* @C_600, in the Sn 3d region (Figure 3a), two main contributions at 487.1 and 495.6 eV were deconvoluted into two lines, indicating the presence of Sn^2+^ and Sn^4+^.^[^ ^48^ ^]^ In both SnS*_x_* @C_600 and SnS*_x_* @C_800 samples, Sn^4+^ is the predominant oxidation state near the surface—an effect commonly observed due to the material's exposure to air.^[^ ^49^ ^]^ In the Sn 3d spectrum of SnS*_x_* @C_800, a minor shoulder is observed at ≈497 eV. A survey scan of the same electrode (see Figure S5a, Supporting Information) reveals Na 1s and Na 2s signals together with the Cu 2p peak from the current collector and weak O 1s and N 1s contributions from surface adsorbates. The shoulder at 497 eV is therefore assigned to the Na KLL Auger emission and not to an additional Sn oxidation state. Depth‐profiling experiments (see Figure S5b,c, Supporting Information) confirm a stronger Sn^2+^ signal beneath the surface, consistent with the presence of tin sulfide. Notably, the relative molar Sn content at the surface is almost twice as high in SnS*_x_* @C_600 compared to SnS*_x_* @C_800. In the S 2p region (Figure 3b), the first main 2p_3/2_ line at 161.5 eV indicates the presence of Sn─S bonds.^[^ ^50^, ^51^ ^]^ Additional peaks at 163.7 and 164.9 eV correspond to C─S bonding, indicating interactions between the carbon matrix and sulfur species.^[^ ^52^ ^]^ It is worth noting that the carbon black additive (5 wt%) can also contain traces of sulfur, thus potentially contributing to the S 2p signals in minor amounts. In addition, a weak feature at 168.2 eV is assigned to the presence of sulfates(VI), likely formed by partial oxidation of the sulfur species when exposed to air. The C 1s spectra region (Figure 3c) shows the principal component at 284.6 eV, ascribed to sp^3^ and sp^2^ C atoms without electronegative substituents. Peaks at higher binding energies are deconvoluted into contributions from C─S and C─O, whereas signals at 287 eV can be linked to O─C═O groups (carboxyl/ester functionalities). In addition, the line with an energy of 290.8 eV can be ascribed to the shake‐up excitation originating from sp^2^ carbon, which is an additional confirmation of the extended π‐electron system in the material.^[^ ^53^ ^]^

**Figure 4:**
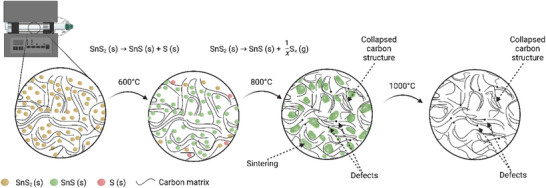
Schematic illustration of the structural and chemical evolution of SnS_2_@C during pyrolysis at 600, 800, and 1000 °C.

Thus, we propose that during the pyrolysis at 600 °C, most of the SnS_2_ is converted to SnS, but the lower temperature may hamper the complete release of sulfur, which instead remains partly retained or trapped within the carbon matrix (see **Figure** **4**). This aligns with the XPS findings (Table S6, Supporting Information), which show comparatively higher surface contents of tin and sulfur for SnS*_x_* @C_600. Some of this residual sulfur may also bond with or become immobilized in the carbon framework. The overall lower temperature likely preserves more micropores and minimizes sintering – an effect consistent with the higher surface area (212 m^2^ g^−1^) for SnS*_x_* @C_600, measured by BET. In Raman spectroscopy, the *I*_D1_/*I*_G_ ratio for SnS*_x_* @C_600 is 1.44, which is unexpectedly lower than the 2.07 ratio for SnS*_x_* @C_800. Although higher pyrolysis temperatures typically yield more graphitic (and thus lower‐defect) carbon, the vigorous loss of sulfur at 800 °C is considered to locally perturb the carbon structure and create additional defects, thereby raising the measured *I*_D1_/*I*_G_ ratio. By 800 °C, the release of sulfur is more effective, consistent with lower Sn and S levels at the electrode surface, yet the ensuing partial sintering and structural rearrangement reduce the surface area (128 m^2^ g^−1^). Overall, the SnS*_x_* @C_800 sample undergoes more thorough sulfur evolution and develops a defect‐rich but compact carbon framework, while SnS*_x_* @C_600 retains extra sulfur in the matrix and consequently exhibits a higher surface area but fewer carbon defects. On the other hand, nearly all residual Sn‐S species volatilize at 1000 °C, leaving behind pores that coalesce into an open micro/mesoporous network. Although the carbon backbone undergoes partial graphitization and “collapses,” the newly created voids contribute to the increased surface area of 321 m^2^ g^−1^ and the pore volume of 0.62 cm^3^ g^−1^. Raman data support this picture: the G band sharpens (HWHM 37 cm^−1^ vs 45 cm^−1^ at 800 °C) and upshifts to 1605 cm^−1^, indicating increased ordering. However, the *I*_D1_/*I*_G_ ratio only falls from 2.07 to 1.84 (still being higher than the 1.44 value at 600 °C) because the newly exposed pore walls introduce fresh edge defects.

**Figure 5:**
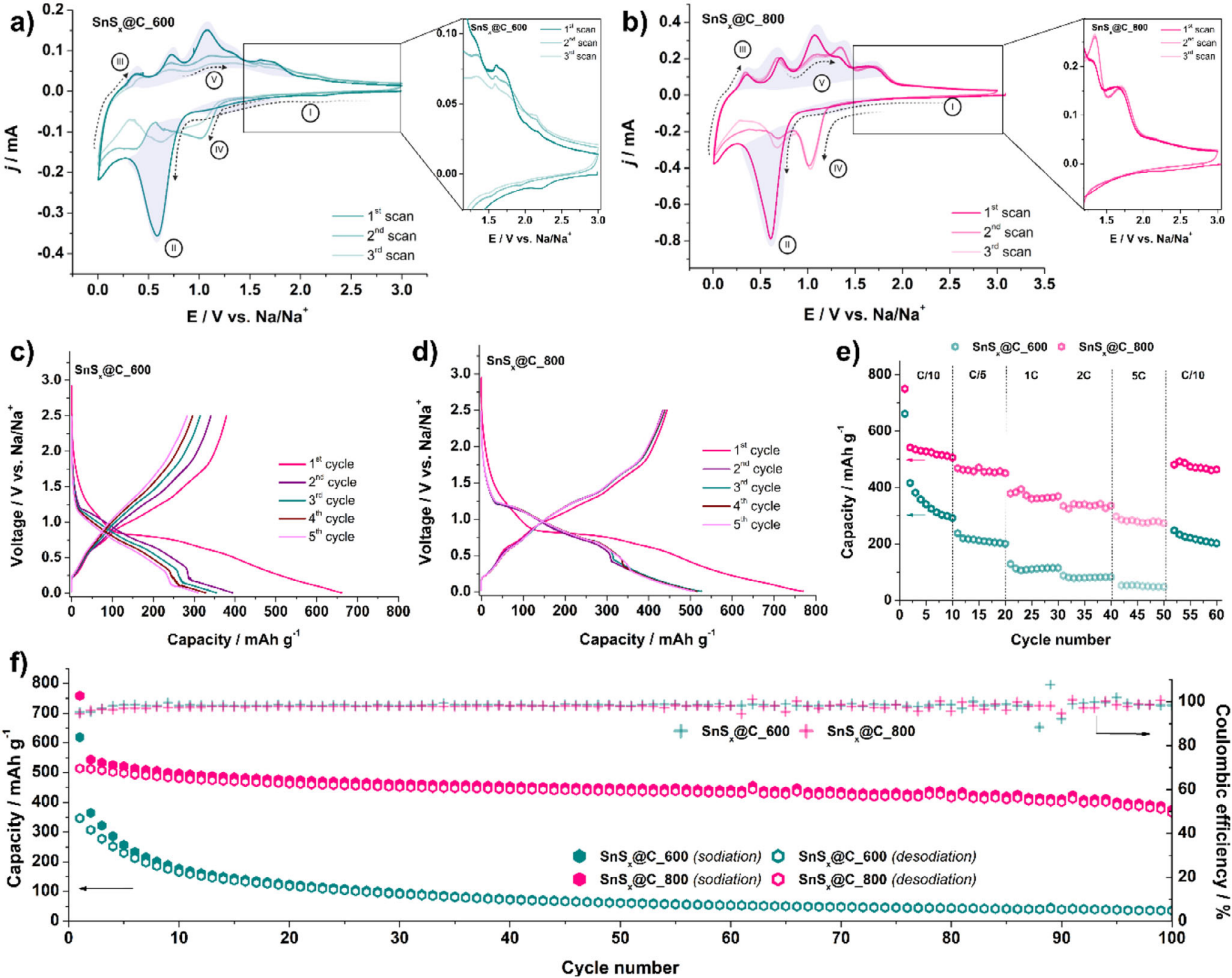
Cyclic voltammograms recorded in 1 m NaPF_6_ in EC:DEC (30:70) + 5% FEC electrolyte (*v* = 100 µV s^−1^) for half‐cells with the a) SnS_x_@C_600 and b) SnS*_x_* @C_800 anode materials; galvanostatic charge–discharge curves recorded in 1 m NaPF_6_ in EC:DEC (30:70) + 5% FEC at current density of *C*/10 for the c) SnS*_x_* @C_600 and d) SnS*_x_* @C_800; e) rate capability tests; f) cycling performance at current density of *C*/10 for the SnS*_x_* @C_600 and SnS*_x_* @C_800 anode materials.

The composites and their behavior during sodium‐ion storage were investigated via cycling voltammetry in 1 m NaPF_6_ in ethyl carbonate:diethyl carbonate (EC:DEC) (30:70) + 5% FEC. The CV curves for the SnS*_x_* @C_1000 material (see Figure S6a, Supporting Information) do not show reactions associated with tin sulfide, which is in line with the characterization results that showed the loss of tin sulfide. For the SnS*_x_* @C_600 material (**Figure** **5**a), distinct yet subtle peaks are found that are not discernible for the materials annealed at higher temperatures. In region I of the first cathodic scan, a small reduction signal at ≈2.2 V is followed by an anodic peak at ≈1.6 V in the reverse scan. Given the XPS results showing residual sulfur species and the Raman data indicating SnS and C─S interactions, these peaks likely correspond to redox processes of surface‐confined sulfur species. The initial cathodic signal suggests the reduction of surface sulfur species, forming sulfur intermediates. The subsequent anodic peak indicates their re‐oxidation. Since these signals are absent in the SnS*_x_* @C_800 material (see Figure 5b), it is reasonable to assume that sulfur sublimation occurs more efficiently at 800 °C, reducing the available amount of the component, causing the redox process. For both SnS*_x_* @C_600 and SnS*_x_* @C_800, an intense peak is distinguished at around 0.56 V during the first negative scan at region II, indicating the conversion of SnS to Na_2_S and metallic Sn, the electrolyte reduction, and the formation of a SEI.^[^ ^39^, ^54^ ^]^ The peak is only observed in the first potential cycle. In the reverse scan and the region III, multiple peaks were registered, including anodic ones at 0.36, 0.72, 1.06, and 1.34 V, with the corresponding reduction peaks in cathodic scan at 0.27, 0.69, and 1.01 V, attributed to the Na‐Sn alloying/dealloying reactions.^[^ ^39^, ^55^ ^]^ For SnS*_x_* @C_600, continuous changes and a decrease in current over successive cycles indicate material degradation. For SnS*_x_* @C_800, the anodic peak at 1.7 V indicates a reversible conversion reaction of Na_2_S back to SnS,^[^ ^56^ ^]^ which is clearly visible and remains stable over cycles. However, in SnS*_x_* @C_600, this peak appears only in the first cycle and diminishes thereafter, reflecting a progressive loss of reversibility and electrochemical activity (see the inset for both Figure 5a,b). Differences in electrochemical performance are further evident in the galvanostatic charge–discharge curves. For SnS*_x_* @C_600 (Figure 5c), a clear decrease in capacity is observed with each cycle, highlighting its limited cycling stability. In contrast, SnS*_x_* @C_800 (Figure 5d) maintains a stable capacity, with the initial coulombic efficiency (ICE) of 68%. On the other hand, the ICE is 56% for SnS_x_@C_600 and 35% for SnS*_x_* @C_1000. The first‐cycle voltage profile of SnS*_x_* @C_1000 (see Figure S6b, Supporting Information) confirms an unusually large irreversible capacity centered above 0.6 V, exactly where extensive SEI formation is expected. This drop in ICE correlates with the BET analysis: the surface area exhibits a step‐increase from 128 m^2^ g^−1^ at 800 °C to 321 m^2^ g^−1^ at 1000 °C, while the pore volume increases from 0.15 to 0.62 cm^3^ g^−1^, providing an enhanced carbon/electrolyte interface that consumes Na^+^ irreversibly in the first cycle. Regarding the effect of pyrolysis temperature on ICE, Li et al.^[^ ^57^ ^]^ have reviewed temperature‐induced graphitization in hard carbons and concluded that raising the carbonization temperature from ≈600 to 1400 °C steadily closes open pores, lowers surface area, and therefore increases ICE. A typical example is paulownia‐derived hard carbon, where the ICE increased from 76.8% at 1000 °C to 85.9% at 1400 °C as the pore volume collapsed and the graphitization degree rose.^[^ ^58^ ^]^ Furthermore, it is generally stated that higher defect density lowers the ICE, because vacancies, edge sites and sp^3^ domains may trap Na^+^ irreversibly. An example is the work by Yang et al.^[^ ^59^ ^]^ focusing on wheat‐starch hard carbon and graphene‐induced graphitized carbons, where lowering the *I*_D_/*I*_G_ ratio and the BET area pushed the ICE up to 90%–94%. Yet, contrary to most of the presented research, it seems that chemically engineered defects can overturn this trend, which was discussed by Wang et al.^[^ ^60^ ^]^ Defect‐rich long‐range graphene nanoribbons were synthesized by forcing N/S heteroatoms to escape at increased pyrolysis temperature. The resulting hard carbon delivered 284.5 mAh g^−1^ with an ICE of 85.9% (doped material), which was significantly lower for the less‐defective sample (ICE: 72.7%, pristine material).^[^ ^60^ ^]^ Density‐functional calculations showed that the presence of vacancies lowers the Na‐adsorption energy and cuts the interlayer diffusion barrier from 0.69 to 0.51 eV, while the same high‐temperature step decreases the BET area, thus limiting SEI formation.^[^ ^60^ ^]^ A similar “beneficial‐defect” pattern was observed for the SnS*_x_* @C_800 electrode material in this study: sulfur volatilization at 800 °C increases the *I*_D_/*I*_G_ ratio to 2.07 (compared to 1.44 at 600 °C) yet simultaneously lowers the surface area by ≈40% and removes sulfur from the matrix. The surface‐area and defect formation effects therefore outweigh the possible Na‐trapping by those additional defects, indicating that the ICE is governed by the balance between defect chemistry and electrolyte interface, and that controlled heteroatom escape is a viable route to enhanced Na‐ion kinetics and higher first‐cycle efficiency in both hard carbon and chalcogenide–carbon composites. Rate capability tests, illustrated in Figure 5e, confirm this trend, showing a continuous capacity decay for SnS*_x_* @C_600 across increasing current densities. On the other hand, SnS*_x_* @C_800 demonstrates superior rate performance, maintaining a capacity of ≈500 mAh g^−1^ at a current density of *C*/10, underscoring the ability of the material to sustain its electrochemical activity, even under varying charge–discharge conditions. Finally, long‐term cycling results, shown in Figure 5f, further emphasize the significant stability advantage of the SnS*_x_* @C_800 material. Over 100 cycles, it retains 73% of its initial capacity, demonstrating robust performance and structural integrity. In contrast, the SnS*_x_* @C_600 material exhibits rapid capacity loss, with a loss of over 82% of its initial capacity within the first 50 cycles. Moreover, average‐voltage hysteresis ($\Delta \bar{V}$, see Table S7, Supporting Information), which was calculated based on the hysteresis curves (see Figure S6c–e, Supporting Information), further confirms the differences between the materials. SnS*_x_* @C_800 delivers the highest reversible capacity, with a low average charge voltage (1.21 V) and a small hysteresis (0.53 V). In contrast, SnS*_x_* @C_600 is characterized by a higher ${\bar{V}}_{\mathrm{chg}}$ of up to 1.30 V, further increasing upon cycling, and rising hysteresis, which points to impedance growth and sluggish desodiation process.^[^ ^61^, ^62^ ^]^ Literature reports suggest that this may be the effect of a continuously growing SEI, which is also consistent with decreased reversibility.^[^ ^63^, ^64^ ^]^ However, it still needs to be borne in mind that in comparison with materials for SIBs, like hard carbons^[^ ^65^ ^]^ or polyanion‐type materials,^[^ ^66^ ^]^ conversion‐alloying materials suffer from high average voltages and hysteresis that still need improvement.^[^ ^67^ ^]^

**Figure 6:**
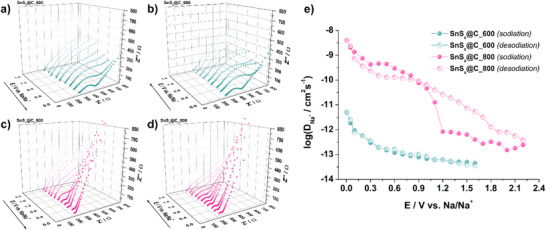
Staircase potentio‐EIS results for a,b) SnS*_x_* @C_600 and c,d) SnS*_x_* @C_800 half‐cells for sodiation (a, c) during desodiation (b, d), focusing on the impedance range up to 800 Ω, and e) calculated diffusion coefficient values in a frequency range of 20 kHz to 1 mHz.

Electrochemical impedance spectroscopy was conducted in staircase potentiostatic mode. The full‐range frequency response is presented in Figure S7 (Supporting Information). The Nyquist plots obtained during the second sodiation and desodiation cycles in a narrower frequency range reveal clear differences between the SnS*_x_* @C_600 (**Figure** **6**a,b) and SnS*_x_* @C_800 (Figure 6c,d) electrodes, providing insight into their charge transfer resistance, sodium‐ion diffusion behavior, and interfacial stability. Each spectrum consists of a high‐to‐medium frequency semicircle, attributed to charge transfer resistance (*R*_ct_) and SEI characteristics, followed by a low‐frequency Warburg‐type linear region, corresponding to sodium‐ion diffusion kinetics. In a Nyquist plot, a Warburg element appears as an inclined line with a 45° slope and is typically described as follows:

(2) $$Z_{W}\;\left(\omega \right)=\sigma \omega^{-1/2}\left(1-j\right)$$

where ω is the angular frequency and σ is the Warburg coefficient. To determine σ, the real part of the impedance was plotted against ω^−1/2^ in the low‐frequency region, and the slope of the linear fit was calculated. Eventually, the diffusion coefficient *D* of the mobile ions can be estimated using:

(3) $$D={\left(\frac{RT}{An^{2}F^{2}c\sigma }\right)}^{2}\;$$

where *R* is the gas constant, *T* the absolute temperature, *A* the electrode area, *n* the charge transferred per ion, *F* the Faraday constant, and *c* the concentration of the diffusing species. In this analysis, the low‐frequency Warburg impedance was attributed primarily to electrolyte diffusion within the electrode pores (rather than solid‐state ion transport in the active material). Consequently, the bulk electrolyte concentration was used in the diffusion coefficient calculations. It should be noted, however, that the actual in‐pore concentration can deviate from the nominal bulk value due to partial depletion and local mass transport effects; hence, the resulting diffusion coefficient reflects an effective or apparent value for electrolyte infiltration under these experimental conditions. Nyquist spectra were fitted with the equivalent circuit (Figure S8, Supporting Information), and the fitted data for every potential step during desodiation for both SnS*_x_* @C_600 and SnS*_x_* @C_800 are presented in Tables S8 and S9 (Supporting Information), respectively. During desodiation, *R*_SEI_ fluctuates between 50 and 100 Ω for SnS_x_@C_600, indicating a less stable SEI that continuously reforms.^[^ ^68^, ^69^ ^]^ On the other hand, *R*_SEI_ only rises from 13 to 81 Ω for SnS*_x_* @C_800, evidencing a thinner and more stable interphase.^[^ ^49^, ^70^, ^71^ ^]^ *R*_ct_ is an order of magnitude lower (see Figure S9, Supporting Information) for SnS*_x_* @C_800 (20–80 Ω) than for SnS*_x_* @C_600 (100–1100 Ω), which is the consequence of a more conductive carbon matrix formed at 800 °C. Moreover, the tenfold smaller Warburg coefficient (12 vs 123 Ω s^−1/2^) indicates faster Na^+^ transport through the partially closed, mesoporous matrix. Calculated sodium‐ion diffusion coefficients further highlight the superior ion transport in SnS*_x_* @C_800 (see Figure 6e). At the beginning of sodiation, the diffusion coefficient for SnS*_x_* @C_800 is 4.0 × 10^−13^ cm^2^ s^−1^, which is an order of magnitude higher than in SnS*_x_* @C_600 (4.5 × 10^−14^ cm^2^ s^−1^). As the sodiation process continues, the diffusion coefficients increase, reaching 4.0 × 10^−9^ cm^2^ s^−1^ at 0.005 V for SnS*_x_* @C_800, compared to 4.9 × 10^−12^ cm^2^ s^−1^ for SnS*_x_* @C_600—a difference of nearly three orders of magnitude. According to the calculated *D*_Na+_, sodium extraction is also kinetically significantly more favorable in the SnS*_x_* @C_800 matrix than for SnS*_x_* @C_600. This result suggests that sodium‐ion insertion and deintercalation is much more efficient for SnS*_x_* @C_800, due to its higher electronic conductivity as a direct consequence of the higher pyrolysis temperature. The striking difference in EIS response between SnS*_x_* @C_600 and SnS*_x_* @C_800 can also be linked directly to their pore architecture. BET analysis shows that SnS*_x_* @C_600 is dominated by open micropores (≈0.5 nm), giving a high external surface area. When electrolyte penetrates this network, SEI is forced to grow throughout it, and the ICE is limited to 56%. However, raising the pyrolysis temperature to 800 °C results in partial sintering while also introducing a moderate 1–4 nm mesopore fraction (see Figure S4b, Supporting Information), with an exposed surface area reduced to 128 m^2^ g^−1^ and an improved ICE (68%). These new mesopores also shorten Na^+^ diffusion paths, explaining the order‐of‐magnitude higher *D*_Na+_. When the pyrolysis temperature was increased to 1000 °C, the volatilized SnS generates an extensive micro/mesoporous network. Although the transport may be kinetically favored, significantly larger electrode/electrolyte interface area results in a thicker SEI and the ICE falls to 35%, limiting its practical application. This is consistent with recent reports indicating that closed micropores enhance ICE, a moderate mesopore fraction may facilitate ion transport, and excessive mesoporosity results in suppressed ICE despite the enhanced kinetics.^[^ ^72^, ^73^, ^74^, ^75^ ^]^

**Figure 7:**
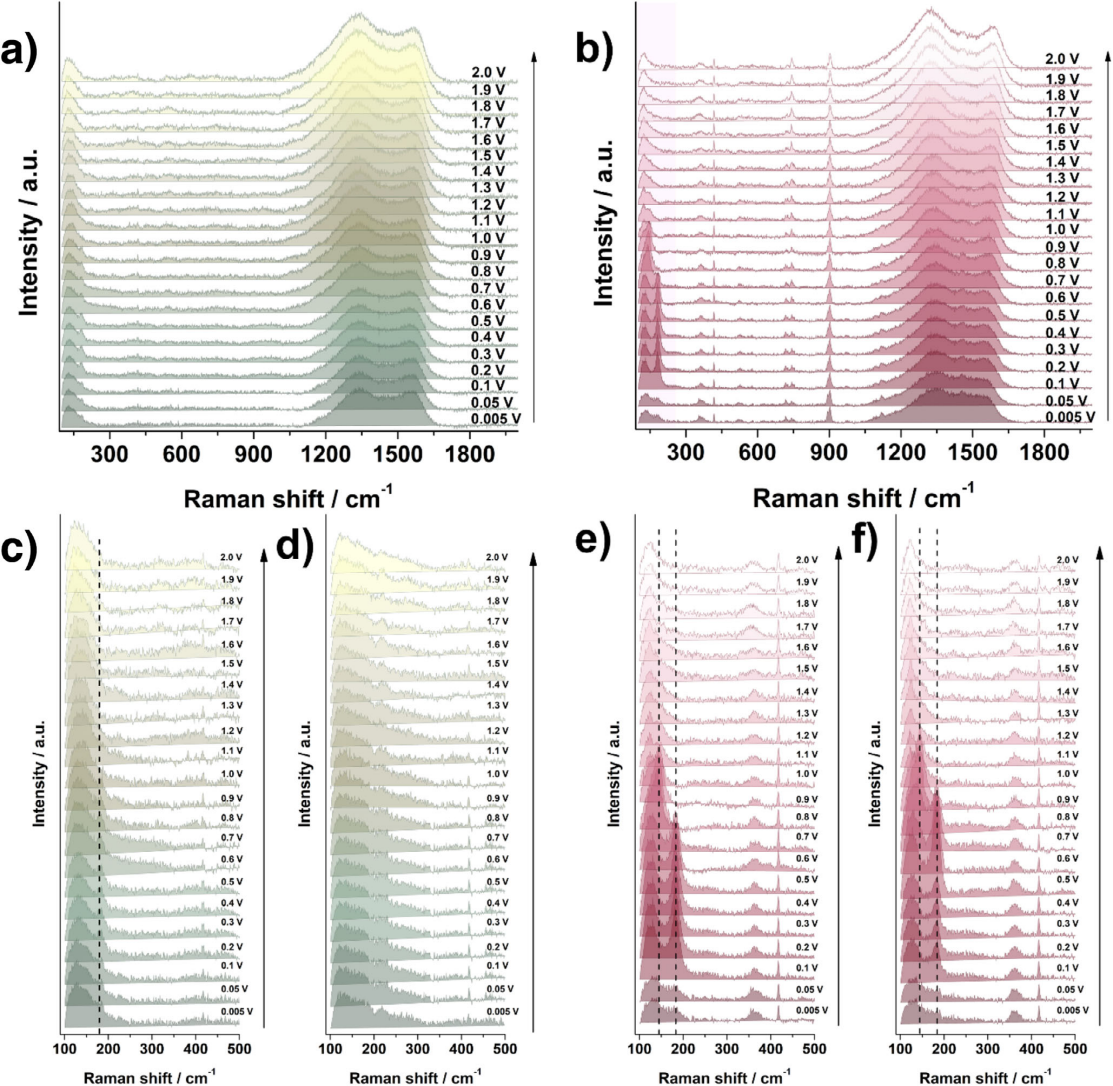
Raman spectra in a full range recorded during desodiation for a) SnS*_x_* @C_600 (2nd cycle) and b) SnS*_x_* @C_800 (2nd cycle); 100–500 cm^−1^ range for c) SnS*_x_* @C_600 (2nd cycle), d) SnS*_x_* @C_600 (20th cycle), e) SnS*_x_* @C_800 (2nd cycle), and f) SnS*_x_* @C_800 (20th cycle).

To gain insight into the behavior of these electrode materials during repeated charge–discharge (sodiation–desodiation) cycles, *operando* Raman spectroscopy measurements were carried out on both samples. The second cycle was examined in detail, along with the 20th cycle, to assess any long‐term changes. The analysis focused on spectral regions indicative of tin sulfide phases and the carbon matrix within the composite. In addition to the main features discussed in this section, several bands in the Raman spectra can be attributed to the electrolyte, e.g., the signals at ≈715 and 892 cm^−1^, corresponding to the O═C ring bending mode and a symmetric ring breathing mode of EC, respectively. The CH_2_─O stretching of DEC is represented by the band at ≈900 cm^−1^, while the band at 742 cm^−1^ describes the symmetric vibration of PF_6_^−^ ions,^[^ ^76^ ^]^ with a weaker band at 417 cm^−1^, typically associated with one of the bending or deformation modes of PF_6_^−^. Representative Raman spectra for the desodiation process in the second scan are presented in **Figure** **7**a,b (full‐range sodiation spectra are provided in Figure S10a,b, Supporting Information).

### 2.1 Second Scan: Tin Sulfide Evolution during Cycling

At 2.0 V, before sodiation, both electrodes show a band at ≈217 cm^−1^, attributed to the presence of tin sulfide (see Figure S10c,e, Supporting Information). Upon sodiation, this band weakens, indicating structural modifications as Na^+^ intercalates. For the SnS*_x_* @C_800 sample, an additional band emerges near ≈180 cm^−1^, which becomes more pronounced at low potentials, confirming the formation of a more sodiated phase (likely Na_2_S‐Sn, or Na*_x_* Sn phases). Notably, this 180 cm^−1^ band is absent in SnS*_x_* @C_600, suggesting an incomplete conversion reaction—an observation consistent with the weaker electrochemical response in this voltage range, as seen in cyclic voltammetry. During the second desodiation scan, the SnS band at 180 cm^−1^ can be distinguished (Figure 7c,e), especially for SnS*_x_* @C_800, for which an additional feature at ≈150 cm^−1^ also appears transiently. This sequential band behavior suggests the formation of multiple partially sodiated tin sulfide intermediates, indicating a more complex reaction pathway compared to SnS*_x_* @C_600. Moreover, in SnS*_x_* @C_800, this low‐energy band persists over a broader voltage range during desodiation compared to sodiation, whereas for SnS*_x_* @C_600, the signal is much weaker, indicating the process is not as effective, leading to a more irreversible reaction mechanism.

### 2.2 Second Scan: Carbon Matrix Evolution: *I*_D1_/*I*_G_ Ratio and Band Shifts

**Figure 8:**
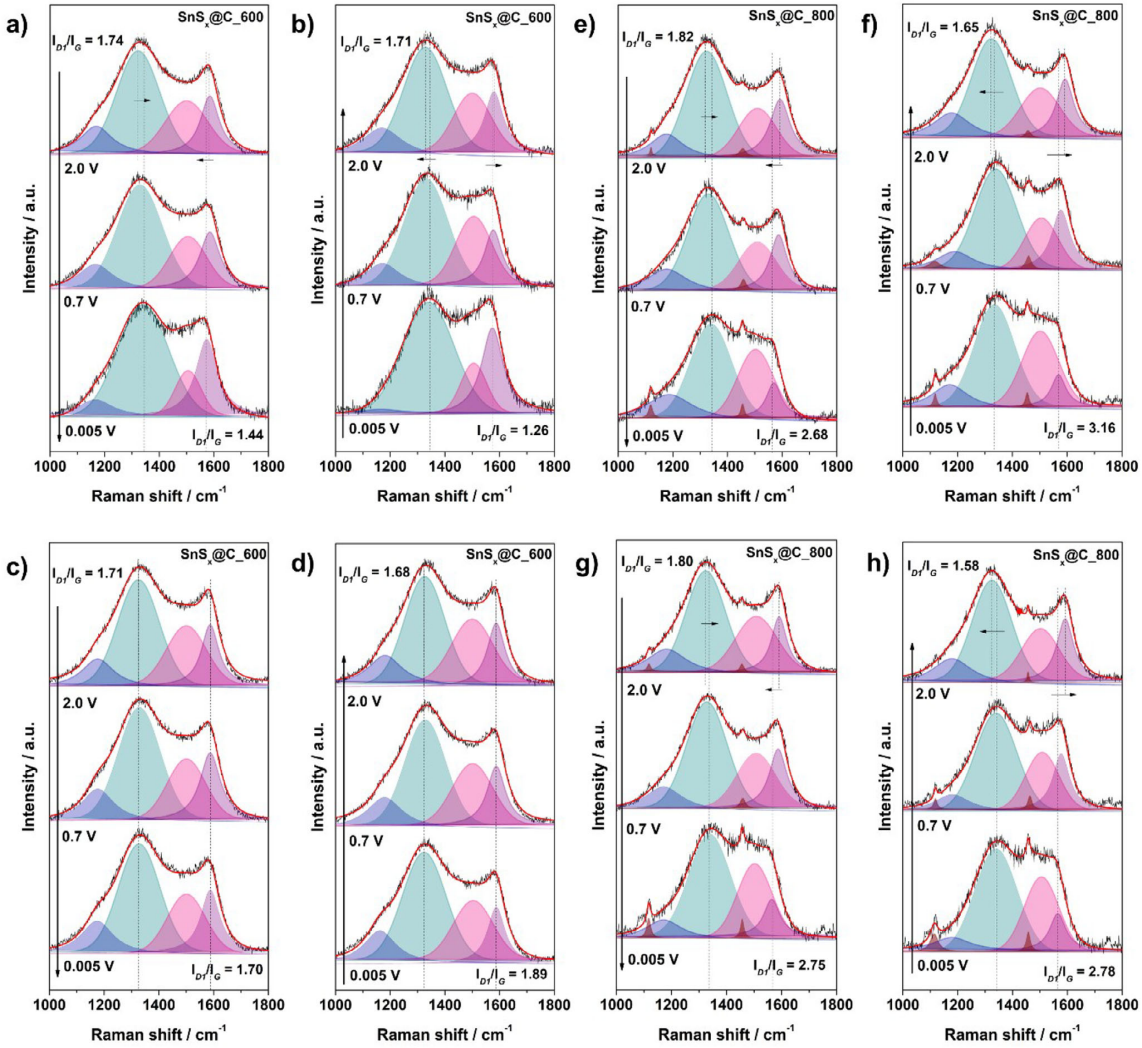
Raman spectra deconvolution for a–d) SnS*_x_* @C_600 and e–h) SnS*_x_* @C_800, spectra recorded *operando* during 2nd sodiation (a,e), 2nd desodiation (b,f), 20th sodiation (c,g), and 20th desodiation (d,h).

In the higher‐wavenumber region associated with the carbon matrix, at first, both samples exhibit shifts in the D1 and G bands upon sodiation, together with variations in band intensity ratio, indicating sodium intercalation into the carbon structure.^[^ ^77^ ^]^ In SnS*_x_* @C_600, the *I*_D1_/*I*_G_ ratio increases from 1.74 at 2.0 V to 1.94 at 0.005 V (**Figure** **8**a and Table S10, Supporting Information), before reversing to 1.71 upon desodiation (Figure 8b and Table S10, Supporting Information). Simultaneously, the G band downshifts from 1585 cm^−1^ at 2.0 V to 1574 cm^−1^ at 0.005 V, then returns to 1579.43 cm^−1^ during desodiation, indicating that Na^+^ intercalation modifies the local bonding environment (reflected by shifts in vibrational modes of the carbon), although the process is largely reversible upon desodiation. For SnS*_x_* @C_800, the spectral shifts are even more pronounced. The *I*_D1_/*I*_G_ ratio increases from 1.82 (2.0 V) to 2.68 (0.005 V) (Figure 8e and Table S11, Supporting Information), before dropping back to 1.65 upon desodiation (Figure 8f and Table S11, Supporting Information). The G band follows a similar trend, shifting from 1592 cm^−1^ (2.0 V) to 1569 cm^−1^ (0.005 V), then restoring to 1592 cm^−1^ after full desodiation. The *I*_D1_/*I*_G_ ratio, which points to edge defects and vacancies, and the ratios *I*_D3_/*I*_G_ and *I*_D4_/*I*_G_, indicating chemical or sp^3^‐type defects, all rise in SnS*_x_* @C_800, but change only modestly (or even fall) in SnS*_x_* @C_600. The large, fully reversible increases in *I*_D3_/*I*_G_ and *I*_D4_/*I*_G_ for the SnS*_x_* @C_800 point to reversible sp^2^ to sp^3^ re‐hybridization, while the smaller response for SnS*_x_* @C_600 indicates that Na^+^ mainly dopes preexisting defects without creating new ones. The greater extent of the shifts for SnS*_x_* @C_800 suggests a more defective carbon structure, which allows stronger interactions with Na^+^. This aligns with previous Raman and BET findings, where higher pyrolysis temperature resulted in more carbon disorder (higher *I*_D1_/*I*_G_ ratio) and a lower surface area, making the carbon more electrochemically active. In contrast, the more microporous, less defective structure of SnS*_x_* @C_600 appears to moderate these interactions, resulting in less intense spectral shifts. Notably, in this wavenumber region, at ≈1115 and 1459 cm^−1^, one or two additional bands are observed, which can be ascribed to signals arising from the EC and DEC vibrations.^[^ ^78^, ^79^ ^]^

### 2.3 Long‐Term Evolution: 20th Cycle Changes

After 20 charge–discharge cycles, the Raman spectra of SnS*_x_* @C_800 remain largely unchanged, retaining clear signals associated with tin sulfide transformations. The SnS‐related band at ≈180 cm^−1^ is still present before sodiation (Figure S10f, Supporting Information). In addition, the transient 150 cm^−1^ feature, which appeared in the second desodiation cycle, persists (Figure 7f), reinforcing the stepwise, controlled nature of Na^+^ insertion and removal. This suggests that SnS*_x_* @C_800 continues to maintain its well‐defined reaction mechanism, supporting its superior electrochemical performance over prolonged cycling. In contrast, significant changes emerge for SnS*_x_* @C_600. The sodiated SnS‐related signals at ≈180 and 150 cm^−1^ become unidentifiable (Figure S10d, Supporting Information), suggesting that sodiation remains incomplete. There is only a broadened, weakened signal at ≈217 cm^−1^, showing the tin sulfide is still there, but does not participate in the sodiation process, and the desodiation spectra are almost identical (Figure 7d), confirming that energy storage is not ongoing.

Moreover, sodium intercalation/deintercalation into the carbon framework also remains active in SnS*_x_* @C_800, reflecting the behavior observed in earlier cycles. The G band shift from 1591 cm^−1^ at 2.0 V to 1565 cm^−1^ at 0.005 V remains significant (Figure 8g and Table S13, Supporting Information), and the back‐shift during deintercalation is repetitive (Figure 8h and Table S13, Supporting Information), confirming that Na^+^ interaction with the carbon matrix remains effective even after prolonged cycling. The graphitization contrast established above persists after 20 cycles. SnS*_x_* @C_800 keeps a G‐band width of 43–45 cm^−1^ and still shows reversible swings in the intensity ratios *I*_D1_/*I*_G_, *I*_D3_/*I*_G_, and *I*_D4_/*I*_G_, confirming that the material is a more ordered sp^2^ framework that continues to intercalate Na^+^ between the layers. In SnS*_x_* @C_600, the G band remains broad and all D band ratios vary insignificantly, indicating that the carbon matrix has become electrochemically inert and Na^+^ storage is now limited to surface sites. These consistent structural changes suggest that SnS*_x_* @C_800 maintains its defect‐rich carbon framework, ensuring long‐term Na^+^ storage capability and structural resilience. Conversely, in SnS*_x_* @C_600, the shifts in D1 and G bands become negligible for both sodiation (Figure 8c and Table S12, Supporting Information) and desodiation (Figure 8d and Table S12, Supporting Information) processes, indicating that the carbon phase is no longer an active participant in the electrochemical process. The *I*_D1_/*I*_G_ ratio undergoes only minimal variation, sharply contrasting the greater response in SnS*_x_* @C_800, suggesting that the carbon network in SnS*_x_* @C_600 has undergone irreversible transformations, limiting its ability to accommodate Na^+^.

All the findings above can be attributed to distinct SEI formation mechanisms, influenced by both the electrode surface composition and its specific surface area. In SnS*_x_* @C_600, the higher tin and sulfur content at the electrode surface and the increased specific surface area (212 m^2^ g^−1^) appear to prevent the establishment of a stable, uniform SEI layer. As repeated sodiation‐desodiation induces volume changes in the tin sulfide, the SEI undergoes partial breakdown and reformation, leading to continuous electrolyte consumption. This ongoing process is amplified by the larger surface area, which provides more active sites for side reactions, accelerating capacity loss and overall degradation. The pronounced decrease in the intensity of electrolyte‐derived Raman signals for SnS*_x_* @C_600 further supports this, as it indicates continuous electrolyte decomposition and incomplete SEI reformation. By contrast, SnS*_x_* @C_800, where the surface area is lower (128 m^2^ g^−1^) and the carbon framework is more defective but structurally stable, a more coherent SEI layer likely forms, effectively passivating the electrode and preventing excessive electrolyte consumption. This allows SnS*_x_* @C_800 to retain distinct tin sulfide signals in Raman spectra even after prolonged cycling, indicating better preservation of the chalcogenide‐based phase. Moreover, the sustained D1/G‐band shifts confirm that its carbon matrix remains electrochemically active, supporting a more stable and reversible Na^+^ storage process.

**Figure 9:**
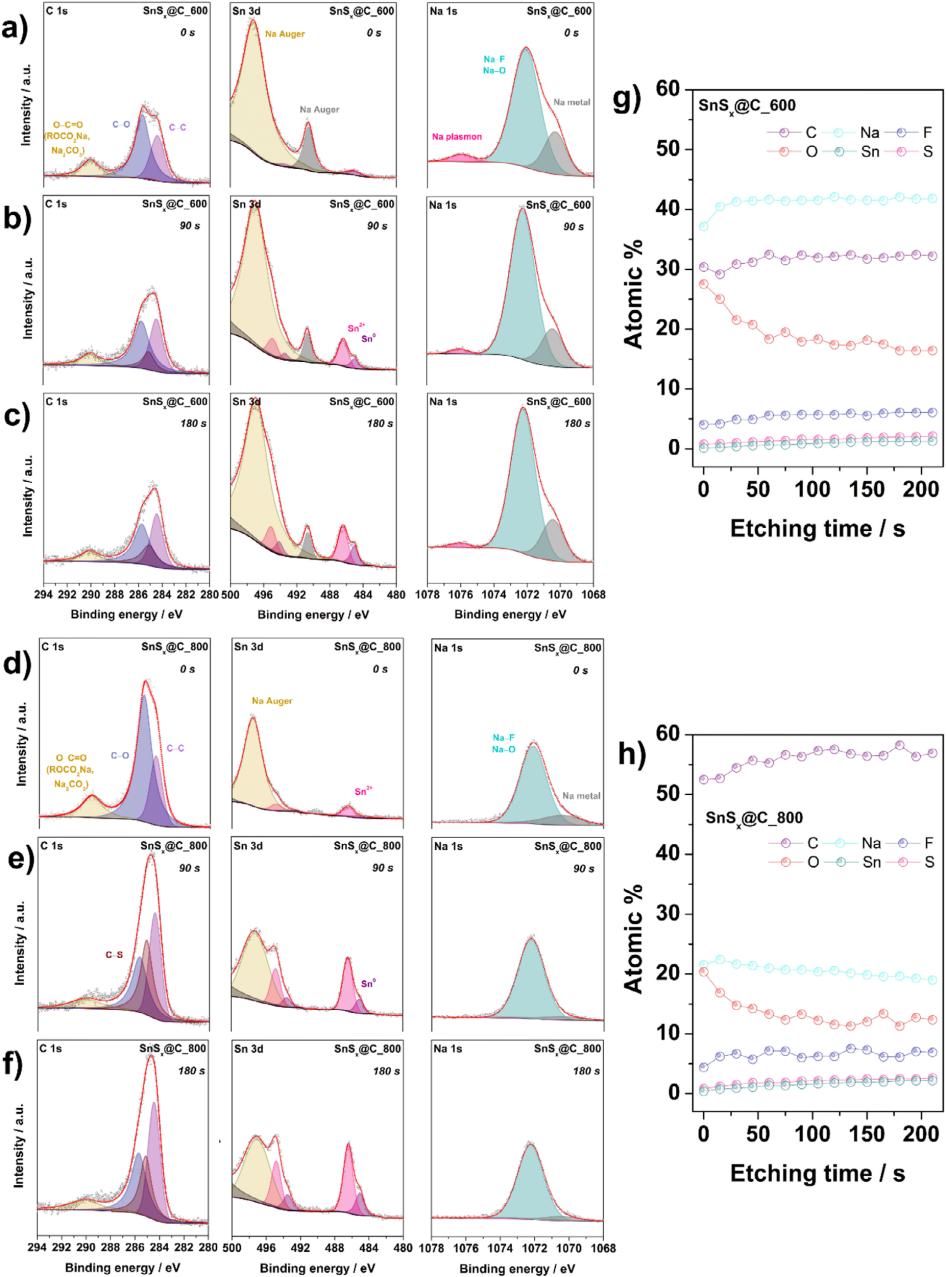
XPS depth‐profiling results for a–c) SnS*_x_* @C_600 and d–f) SnS*_x_* @C_800 at (a,d) 0 s, (b,e) 90 s, and (c,f) 180 s of Ar^+^ ion sputtering with the corresponding relative elemental composition profiles for g) SnS*_x_* @C_600 and h) SnS*_x_* @C_800.

Electrodes were analyzed using XPS depth profiling after 20 charge–discharge cycles, studied in the desodiated state. By examining the surface and subsurface regions (**Figure** **9**a–c for SnS*_x_* @C_600 and Figure 9e–g for SnS*_x_* @C_800), the spectra were obtained before (0 s) and after 90 and 180 s sputtering time. Additional spectral regions (O 1s, F 1s, and S 2p) are displayed in Figure S11 (Supporting Information). The goal was to identify changes in chemical composition and potential interfacial reactions occurring during cycling. Prior to depth profiling, electrodes were washed to remove residual electrolyte salts.

Initially (before sputtering, designated as 0 s), both SnS*_x_* @C_600 and SnS*_x_* @C_800 display pronounced peaks at 285.7 and 290.1 eV in the C 1s region, corresponding to C═O and O─C═O species, respectively (Figure 9a,e). These features are commonly associated with inorganic carbonates (Na_2_CO_3_) and sodium alkyl carbonates (ROCO_2_Na, O─C), indicating a surface SEI layer rich in oxygen‐containing compounds.^[^ ^80^ ^]^ After sputtering (90 and 180 s), the relative intensities from these oxygenated species significantly decrease, while the C─C peak at 284.4 eV grows (Figure 9b,c,f,g). The simultaneous drop in the O 1s signal at 532.2 eV (Figure S11, Supporting Information) further confirms that a major portion of the organic or carbonate‐based SEI is etched away, exposing a higher fraction of the underlying carbonaceous matrix. In this region, a distinct peak is also observed at ≈528 eV, which corresponds to the shifted Na KLL Auger signal, typically expected around 533 eV.

Interestingly, SnS*_x_* @C_800 retains ≈50 at% carbon at deeper sputtering levels, whereas SnS*_x_* @C_600 shows only ≈30 at% C. In addition, a C─S component—which had been observed in the pristine electrodes—is visible, suggesting a preservation of the carbon–chalcogen framework.^[^ ^52^ ^]^ Analysis of the Sn 3d region reveals a larger fraction of metallic Sn and Sn^2+^ in SnS*_x_* @C_800, while in SnS*_x_* @C_600, a peak at ≈489.9 eV is also observed, which corresponds to a shifted Na KLL Auger signal from metallic sodium. In addition, a Na 1s signal at ≈1070.3 eV is detected, which also indicates the presence of metallic sodium,^[^ ^81^, ^82^ ^]^ hinting at more pronounced sodium incorporation and conversion reaction that is not fully reversible. Furthermore, the Na 1 s peak at around 1072.1 eV, typically attributed to Na^+^ salts, indicated the presence of sodium fluoride or related fluoride‐based species in both samples,^[^ ^83^ ^]^ which is in agreement with the F 1s signal at 685 eV (Figure S11, Supporting Information). Both Na and F content increase with the etching time, which is consistent with the established multilayer SEI model, where the outer layer is predominantly composed of various organic species (e.g., ROCO_2_Na) generated by electrode instability and degradation during cycling, while the inner layer mainly contains inorganic compounds such as NaF.^[^ ^83^, ^84^ ^]^ Also, the overall sodium content is nearly twice as high in the SnS*_x_* @C_600 electrode, even after 180 s of sputtering. This aligns with more extensive irreversible Na trapping or side reactions in the SnS*_x_* @C_600 material. Such observations corroborate the *operando* Raman findings, where SnS*_x_* @C_800 showed more effective and reversible sodiation. The XPS data imply that a thicker, more heterogenous SEI is formed on SnS*_x_* @C_600, with a higher proportion of organic byproducts and residual Na^+^. In contrast, SnS*_x_* @C_800 exhibits a relatively thinner and more carbon‐dominated near‐surface region. Moreover, although bulk‐averaged EDS (Tables S1–S4, Supporting Information) confirms the near‐stoichiometric Sn/S ratio of both SnS*_x_* @C_600 and SnS*_x_* @C_800, the surface‐sensitive XPS data reveal that SnS*_x_* @C_600 exposes more S sites than SnS*_x_* @C_800. This surface enrichment promotes a thicker SEI and the higher *R*_SEI_ observed by EIS, whereas the lower coverage in SnS*_x_* @C_800 favors a thinner, more stable interphase and improved cycling stability. A thicker or more heterogeneous SEI (as implied for SnS*_x_* @C_600) can lead to irreversible capacity losses and compromised ion transport.^[^ ^85^ ^]^ Altogether, the XPS depth profiling clarifies why the SnS*_x_* @C_800 electrode exhibits greater stability and reversibility: its near surface region contains fewer irreversibly bound sodium species, a more robust carbon–sulfur framework signal, and a higher fraction of stable inorganic Sn‐containing phases—all of which promote a more favorable electrode–electrolyte interface during prolonged cycling.

## 3 Conclusion

In summary, this work demonstrates the pivotal influence of pyrolysis temperature on the structure, interfacial chemistry, and electrochemical performance of SnS*_x_* @C anodes for sodium‐ion batteries. By systematically comparing samples carbonized at 600, 800, and 1000 °C, it was shown that the pyrolysis at 800 °C promotes more effective sulfur release from SnS_2_ and yields a defect‐rich yet robust carbon matrix, resulting in a stable electrode–electrolyte interface. *Operando* Raman spectroscopy revealed that SnS*_x_* @C_800 maintains reversible Sn─S redox processes and Na^+^ intercalation throughout extended cycling, whereas the SnS*_x_* @C_600 sample retains extra sulfur in the matrix, hindering complete conversion reactions and leading to capacity decay. XPS depth profiling further confirms that the 800 °C composite forms a thinner SEI layer with fewer irreversibly bound Na species. As a result, SnS*_x_* @C_800 achieves a stable capacity of ≈500 mAh g^−1^ at *C*/10 and retains a substantial fraction of this capacity over 100 cycles, significantly outperforming its lower‐temperature counterpart. These findings highlight the crucial balance between carbon defects, surface area, and SEI formation for optimizing Na^+^ storage performance. Overall, the *operando* spectroscopic insights gained here underscore the importance of tailoring thermal treatments to design stable chalcogenide‐carbon anodes, paving the way toward high‐performance sodium‐ion batteries.

## 4 Experimental Section

### SnS_x_@C Synthesis

The SnS*_x_* @C electrode materials were prepared as follows: 4.72 g of starch ((C_6_H_10_O_5_)*_n_*, Sigma‐Aldrich, USA) and 2.98 g of thioacetamide (C_2_H_5_NS, Chemat, Poland) were mixed with 100 mL of ethylene glycol (C_2_H_6_O_2_, POCH, Poland) under continuous stirring. Subsequently, 3.4 mL of tin(IV) chloride (POCH, Poland) was added to the mixture, and finally, the solution was placed in the Teflon‐lined autoclave at 150 °C for 20 h. The resulting suspension was centrifuged at 9000 rpm with acetone (POCH, Poland) until clear phase separation was achieved. The as‐obtained solid fraction was preliminarily dried at 60 °C for 24 h, and then subjected to annealing under an Ar atmosphere in a quartz tube furnace with a vacuum system. Powders were heated from room temperature to the target pyrolysis temperature at a heating rate of ≈1.7 °C min^−1^, with a holding time of 4 h at the final temperature. Pyrolysis temperatures of 600, 800, and 1000 °C were selected to probe three distinct regions: below the onset of significant Sn/S volatilization, the region where SnS does not yet sublime intensively, and an upper limit where SnS reaches a high vapor pressure and is effectively removed. For brevity, the samples are named as SnS*_x_* @C_600, SnS*_x_* @C_800, and SnS*_x_* @C_1000 depending on the pyrolysis temperature applied. The obtained samples were ground in a mortar and subsequently in a ball mill (Rocker Mill MM 400, Retsch, Germany), with a final step of sieving down to <40 µm. The scheme of materials preparation is presented in Figure S1 (Supporting Information).

### Electrode Preparation

Electrodes for electrochemical testing were prepared by mixing the active material with styrene‐butadiene rubber (SBR, Zeon, Japan), carboxymethylcellulose (CMC, Sigma‐Aldrich, USA), and carbon black (CB, TIMCAL Super P Conductive, Switzerland) in the composition presented in **Table** **2**. The chemicals were mixed with water using an Ultra Turrax (IKA‐Werke, GmbH, Germany). After that, the electrodes were printed on a copper foil using a doctor blade technique (gap distance 120 µm) and dried at 40 °C for 24 h. Finally, the round‐shaped electrodes with a diameter of 10 mm were cut with a cutter and dried in a vacuum oven (B‐585 BUCHI Glass Oven, Switzerland) at 80 °C for 24 h. The weight of the materials on individual electrodes was 2.15 ± 0.35 mg cm^−2^.

**Table 2:** SnS*_x_* @C‐based slurry composition used for electrode printing.

Material	Wt%	Composition
SnS*_x_* @C	85	Powder
Styrene‐butadiene rubber	5	40 wt% in H_2_O/EtOH (vol. 3:7)
Carboxymethylcellulose	5	5 wt% in H_2_O
Carbon Black	5	Powder

### Materials Characterization

Scanning electron microscopy (SEM) was applied for morphological analysis of powders with an accelerating voltage of 30 kV (type 1430 VP, LEO Electron Microscopy Ltd., equipped with EDX). High‐resolution TEM images were recorded using a JEOL JEM‐21000F field‐emission microscope.

N_2_ sorption analysis was performed using ASAP2020 Plus (Micromeritics) after outgassing in a vacuum at 200 °C for 24 h. The specific surface area (*S*_BET_) value was obtained through calculations by the Brunauer–Emmett–Teller equation, whereas the density functional theory (DFT) method was applied to determine the pore size distribution. The total pore volume (*V*_t_) was measured at a single point at the maximum relative pressure (*p*/*p*_0_).

XRD patterns were recorded for powders with the diffractometer (Philips X'Pert) equipped with Cu Kα radiation detector (X'Celerator Scientific, λ = 0.15406 nm).

Raman measurements were performed *operando* by means of Micro‐Raman spectrometer (Renishaw InVia) equipped with a 785 nm diode laser (100 mW). Spectra were recorded through a quartz window of an electrochemical cell using a 50× objective lens. Raman spectra were recorded in two regimes by measuring the entire spectra in the range 120–3200 cm^−1^ and by fast measurement in a narrow spectral range, which enabled shortening the recording time of a single spectrum during electrochemical analysis.

XPS analysis was performed with an ESCALAB 250Xi spectrometer (ThermoFisher, GB), equipped with a monochromized Al Kα source (*E*_h_ *_v_* = 1486.68 eV) set to 650 µm spot size. The standard lens mode was used with a pass energy of 100 eV for survey and 10 eV for high‐resolution spectra. In‐lens charge compensation was applied for samples with insulating SEI. For depth profiling, samples were etched with a MAGCIS Ar ion source (ThermoFisher, GB) in ion mode set at 3 keV and 1.5 mm spot size in cycles of 15 s. Prior to XPS depth profiling, the cycled electrodes were washed to remove residual electrolyte salts. The washing procedure involved immersing each electrode in fresh dimethyl carbonate (DMC, anhydrous, ≥99%, Sigma‐Aldrich, USA) for 5 min. This step was repeated two additional times with fresh portions of DMC, for a total of three washing cycles. To remove remains of organic solvents, electrochemically modified samples were pretreated by etching with the MAGCIS Ar ion source in cluster mode four times for 15 s at 6 keV. For data analysis, Avantage 5.9931 was used.

For *operando* measurements, the ECC‐Opto‐10 cell (EL‐Cell GmbH, Germany), allowing for side‐by‐side arrangements of the electrodes, was used with a galvanostat/potentiostat BioLogic SP‐150. Apart from *operando* Raman analysis, electrochemical measurements were performed in a two‐electrode Swagelok configuration, in 1 m NaPF_6_ in EC/DEC (3:7 by wt%) supplemented with 5 wt% fluoroethylene carbonate (FEC) addition (E‐Lyte, Germany). The prepared materials were used as a working electrode, and a sodium disc (AOT, China) was used as a combined counter and reference electrode, separated by a glass fiber separator (Schleicher&Schill, Germany). The galvanostatic charge/discharge was performed in the potential range from 0.005 to 2.5 V versus Na/Na^+^ with a current density based on the theoretical capacity of graphite, i.e., 1 C = 372 mA g^−1^ (fully charged/discharged in 1 h), e.g., C/10 = 37.2 mA g^−1^. Staircase potentiostatic electrochemical impedance spectroscopy (SPEIS) was conducted in a three‐electrode Swagelok cell with two sodium discs working as counter and reference electrodes, with an amplitude of 10 mV in a frequency range of 20 kHz to 1 mHz. SPEIS measurements were performed at a series of 100 mV potential increments during the charging and discharging of the material. Prior to each impedance measurement, a stabilization period of 4 h was employed to ensure that the system reached steady‐state current conditions, minimizing transient effects and enabling accurate impedance data acquisition. All experiments were performed using the galvanostat/potentiostat BioLogic VMP3 and BioLogic VSP 2078.

## Conflict of Interest

The authors declare no conflict of interest.

## Supporting information



Supporting Information

## Data Availability

The data that support the findings of this study are available from the corresponding author upon reasonable request.
