# Supplementary material for: A Key to Material's Stability: Tuning Pyrolysis Temperature in SnS x @C Anodes for Sodium‐Ion Batteries
Source: Small. 2025 Jul 31;21(38):e04485. doi: 10.1002/smll.202504485 (PMC12462594; doi:10.1002/smll.202504485)
Supplement: Supplementary file 1 — Supporting Information [file SMLL-21-e04485-s001.docx]

Supporting Information

**A Key to Material’s Stability: Tuning Pyrolysis Temperature in SnS*_x_*@C Anodes for Sodium-Ion Batteries**

*Zuzanna Zarach^*^, Mirosław Sawczak, Carsten Dosche, Konrad Trzciński, Mariusz Szkoda, Magdalena Graczyk-Zając, Ralf Riedel, Gunther Wittstock, Andrzej P. Nowak*


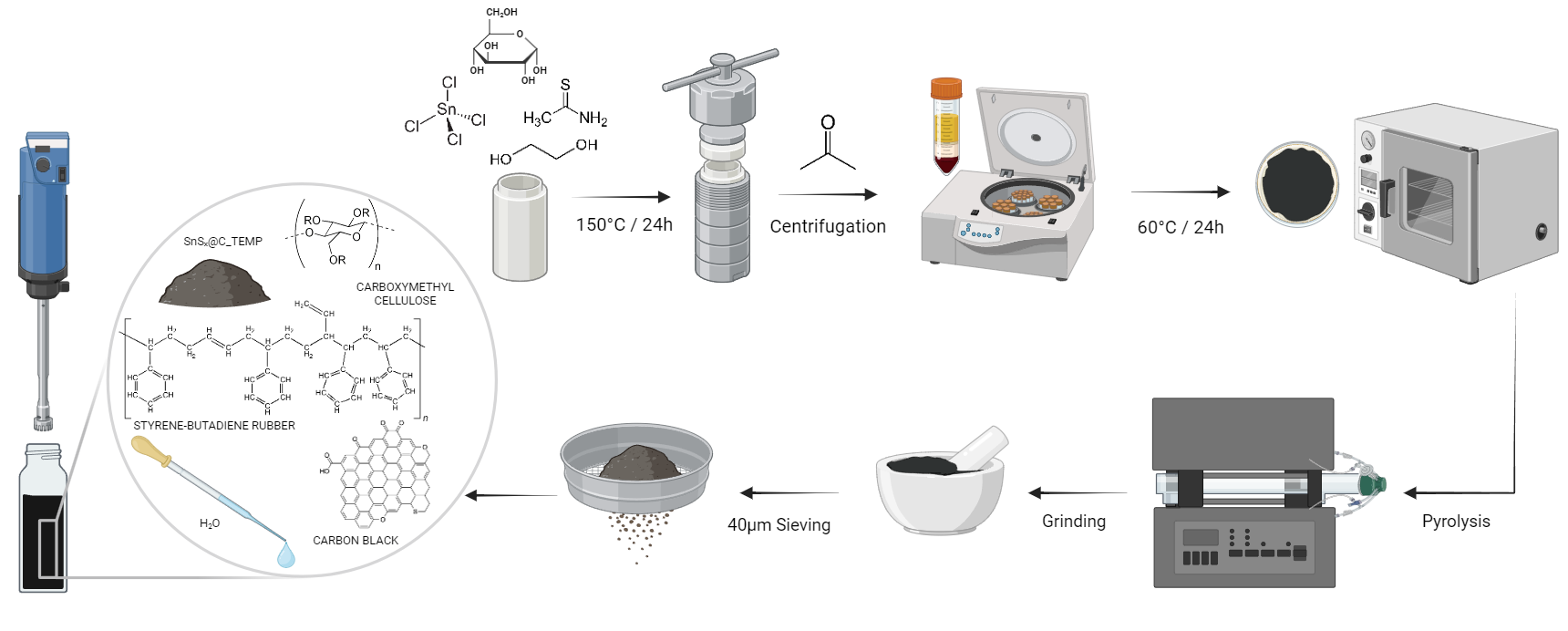


**Figure S1.** Scheme of the SnS*_x_*@C electrode materials preparation.

**Table S1.** EDS analysis for the SnS*_x_*@C-600 powder

| ***SnS_x_@C-600*** | Mass fraction (%) | | | | | |
| --- | --- | --- | --- | --- | --- | --- |
| Spectrum | **C** | **O** | **Al** | **S** | **Cl** | **Sn** |
| Mean Value | 10.75 | 47.75 | 1.80 | 8.93 | 0.13 | 30.55 |
| Sigma | 1.38 | 1.32 | 0.47 | 0.53 | 0.01 | 1.71 |
| Sigma mean | 0.69 | 0.66 | 0.23 | 0.27 | 0.00 | 0.85 |
|  | **Relative elemental composition (%)** | | | | | |
| Value | 19.95 | 66.53 | 1.49 | 6.21 | 0.08 | 5.74 |

**Table S2.** EDS analysis for the SnS*_x_*@C-800 powder

| ***SnS_x_@C-800*** | Mass fraction (%) | | | | |
| --- | --- | --- | --- | --- | --- |
| Spectrum | **C** | **O** | **Al** | **S** | **Sn** |
| Mean Value | 7.50 | 41.85 | 2.14 | 10.72 | 37.79 |
| Sigma | 4.72 | 13.61 | 0.54 | 3.07 | 14.71 |
| Sigma mean | 2.36 | 6.80 | 0.27 | 1.53 | 7.36 |
|  | **Relative elemental composition (%)** | | | | |
| Value | 15.72 | 65.85 | 2.00 | 8.42 | 8.01 |

**Table S3.** EDS analysis for the SnS*_x_*@C-1000 powder

| ***SnS_x_@C-1000*** | Mass fraction (%) | | | | | |
| --- | --- | --- | --- | --- | --- | --- |
| Spectrum | **C** | **O** | **Al** | **Si** | **S** | **Sn** |
| Mean Value | 40.98 | 51.43 | 1.18 | 0.20 | 4.30 | 1.90 |
| Sigma | 1.46 | 1.38 | 0.10 | 0.01 | 0.06 | 0.09 |
| Sigma mean | 0.73 | 0.69 | 0.05 | 0.01 | 0.03 | 0.05 |
|  | **Relative elemental composition (%)** | | | | | |
| Value | 49.97 | 47.08 | 0.64 | 0.11 | 1.97 | 0.23 |

**Table S4.** EDS analysis for the SnS*_x_*@C powder

| ***SnS_x_@C*** | Mass fraction (%) | | | | | |
| --- | --- | --- | --- | --- | --- | --- |
| Spectrum | **C** | **O** | **Al** | **S** | **Cl** | **Sn** |
| Mean Value | 17.72 | 58.76 | 0.76 | 8.00 | 2.37 | 12.39 |
| Sigma | 8.01 | 6.76 | 0.22 | 4.76 | 0.96 | 9.56 |
| Sigma mean | 3.27 | 2.76 | 0.09 | 1.94 | 0.39 | 3.90 |
|  | **Relative elemental composition (%)** | | | | | |
| Value | 26.36 | 65.62 | 0.51 | 4.46 | 1.19 | 1.86 |


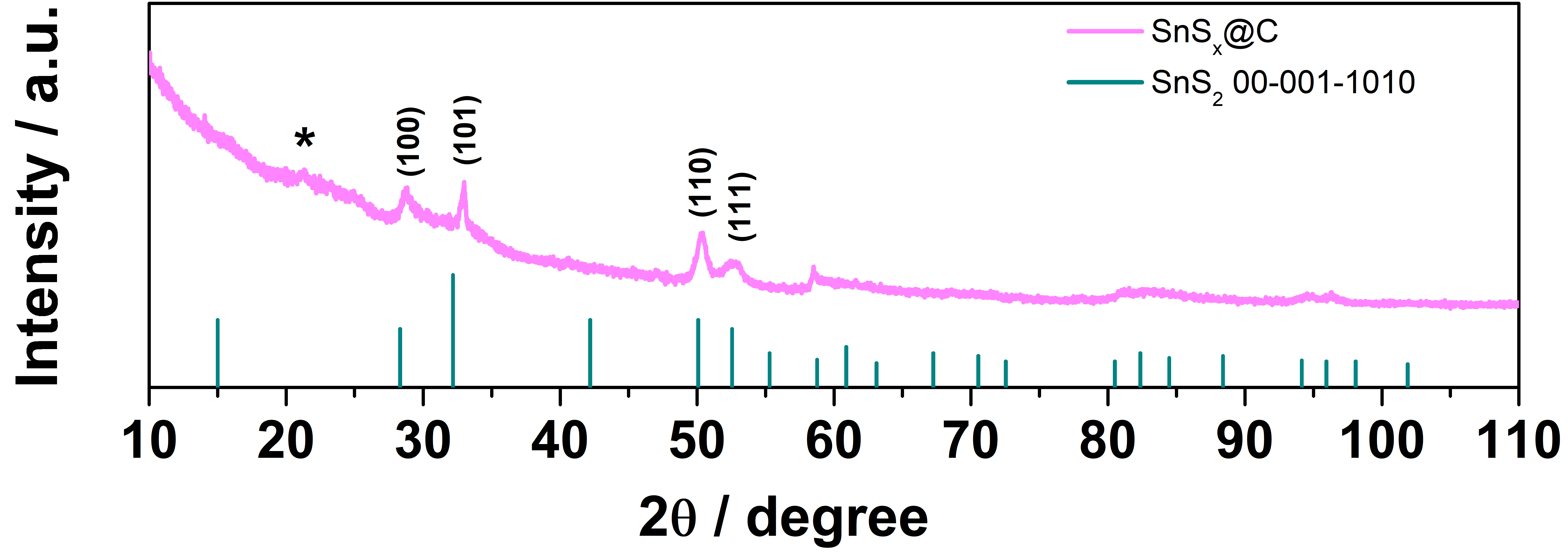


**Figure S2.** XRD patterns for the SnS*_x_*@C powder (without pyrolysis) in the full range.

**
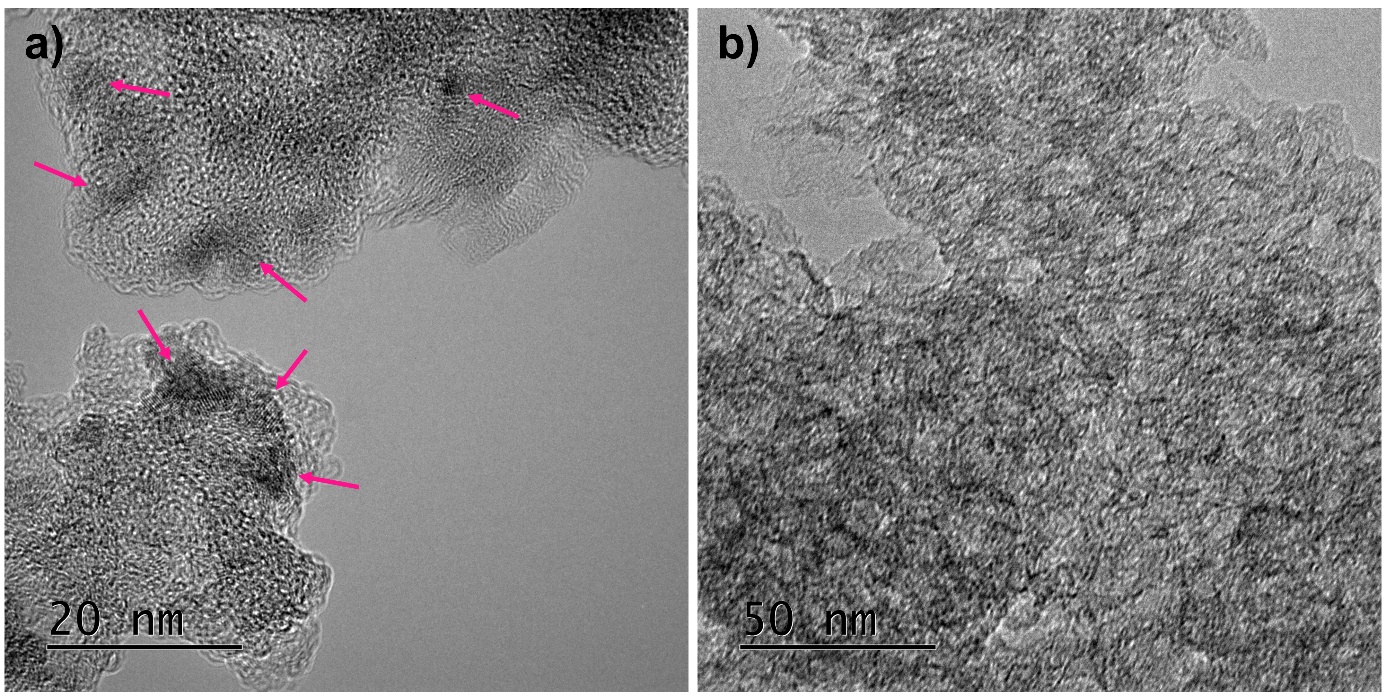
**

**Figure S3.** TEM images recorded for a) SnS*_x_*@C_800 and b) SnS*_x_*@C_1000.


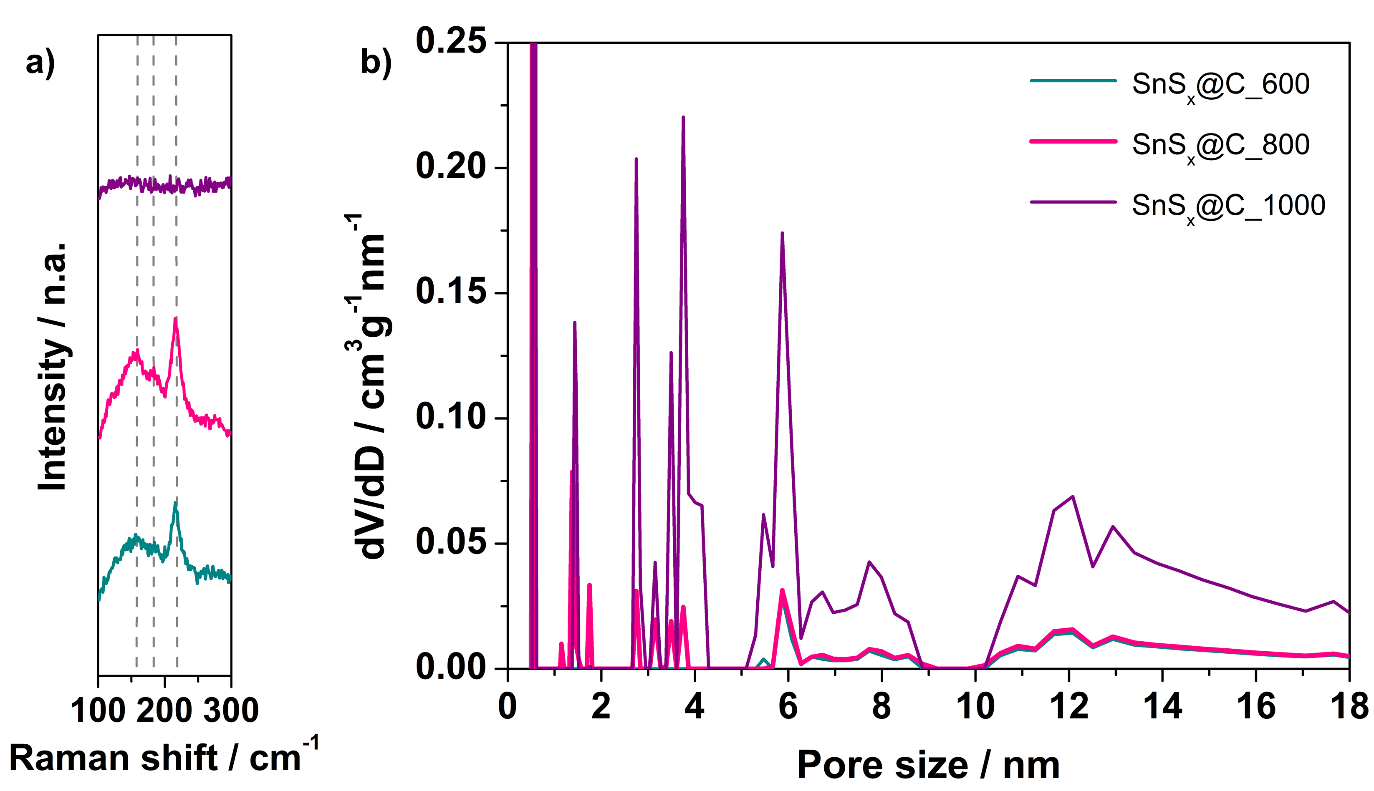


**Figure S4.** a) Raman spectroscopy (short range) for the SnS*_x_*@C powders (pyrolyzed at different temperature) and b) pore size distribution calculated from N_2_ adsorption isotherms based on DFT method for SnS*_x_*@C materials.

**Table S5.** Spectral parameters for the first-order Raman bands of SnS*_x_*@C_600, SnS*_x_*@C_800 and SnS*_x_*@C_1000 electrode materials: band position (Stokes Raman Shift), half width at half maximum (HWHM), and **peak intensity ratios** relative to the G band.

| Band | Spectral parameter | SnS*_x_*@C_600 | SnS*_x_*@C_800 | SnS*_x_*@C_1000 |
| --- | --- | --- | --- | --- |
| G | Position (cm^-1^) | 1590.28 | 1601.51 | 1605.29 |
|  | HWHM (cm^-1^) | 50.16 | 45.13 | 37.12 |
| D1 | Position (cm^-1^) | 1337.57 | 1324.55 | 1327.01 |
|  | HWHM (cm^-1^) | 87.13 | 87.42 | 92.01 |
|  | **I_D1_/I_G_** | **1.44** | **2.07** | **1.84** |
| D3 | Position (cm^-1^) | 1500.33 | 1510.76 | 1525.13 |
|  | HWHM (cm^-1^) | 83.62 | 95.99 | 79.87 |
|  | **I_D3_/I_G_** | **0.77** | **1.11** | **0.77** |
| D4 | Position (cm^-1^) | 1194.39 | 1172.27 | 1173.77 |
|  | HWHM (cm^-1^) | 101.02 | 90.76 | 105.01 |
|  | **I_D4_/I_G_** | **0.74** | **0.86** | **0.42** |

**Table S6.** Elemental composition (atomic %) of the SnS*_x_*@C_600 and SnS*_x_*@C_800 electrodes’ surfaces (before cycling) determined by XPS.

| Element | | Sn | S | C | O | N |
| --- | --- | --- | --- | --- | --- | --- |
| Atomic % | SnS*_x_*@C_600 | 1.34 | 1.77 | 80.47 | 13.79 | 2.64 |
|  | SnS*_x_*@C_800 | 0.64 | 1.08 | 79.16 | 17.75 | 1.36 |

**Impurities analysis**

EDS (**Table S1**–**S4**) and XPS spectra (**Figure 3**, **Figure S5** and **Table S6**) provide both bulk- and surface-sensitive information. Before pyrolysis, the precursor composite (SnS_2_@C) contained ~ 1.2 % Cl, originating from the tin(IV) chloride used as tin precursor. After pyrolysis at 600 °C, the chloride signal drops by more than one order of magnitude (~ 0.08 %), and is already below the XPS detection limit (and was not detected in the other samples). Al was also detected in EDS analysis for every sample, but does not show up in the corresponding XPS survey, even after mild Ar^+^ sputtering, indicating that it originates from the aluminum SEM table and is not incorporated into the powder itself. Finally, a weak Si signal (0.11 %), detected in EDS for the SnS*_x_*@C_1000 material originate from the quartz boat contamination at the highest temperature. None of these elements forms a crystalline phase detectable by XRD, none participates in the electrochemical reactions followed in the paper, and their summed concentration never exceeds 0.3 at.% in the samples that were thoroughly evaluated (SnS*_x_*@C_600 and SnS*_x_*@C_800).


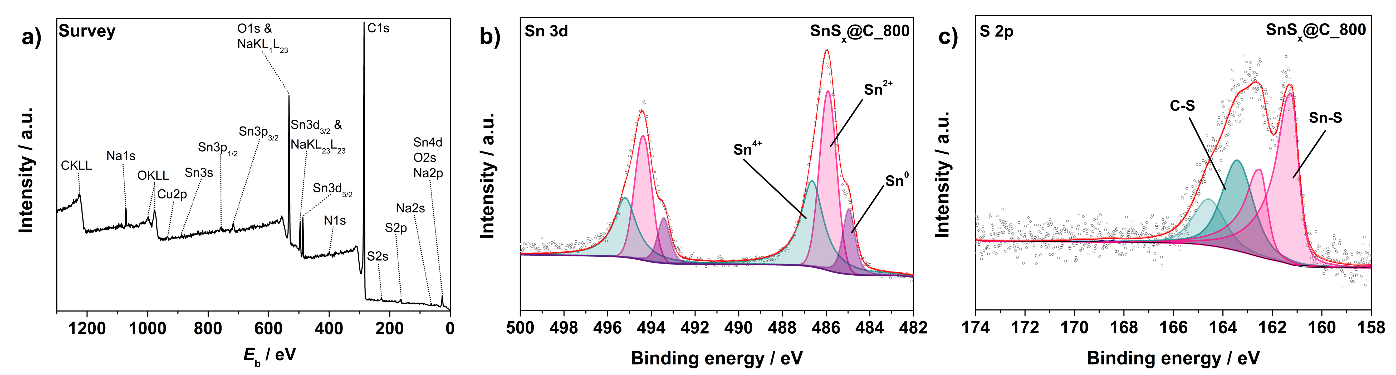


**Figure S5.** XPS (a) survey spectra of SnS*_x_*@C_800 electrode; (b) high-resolution spectrum of Sn 3d and (c) high-resolution spectrum of the S 2p regions for the SnS*_x_*@C_800 electrode materials after 60 s etching with Ar^+^.

*
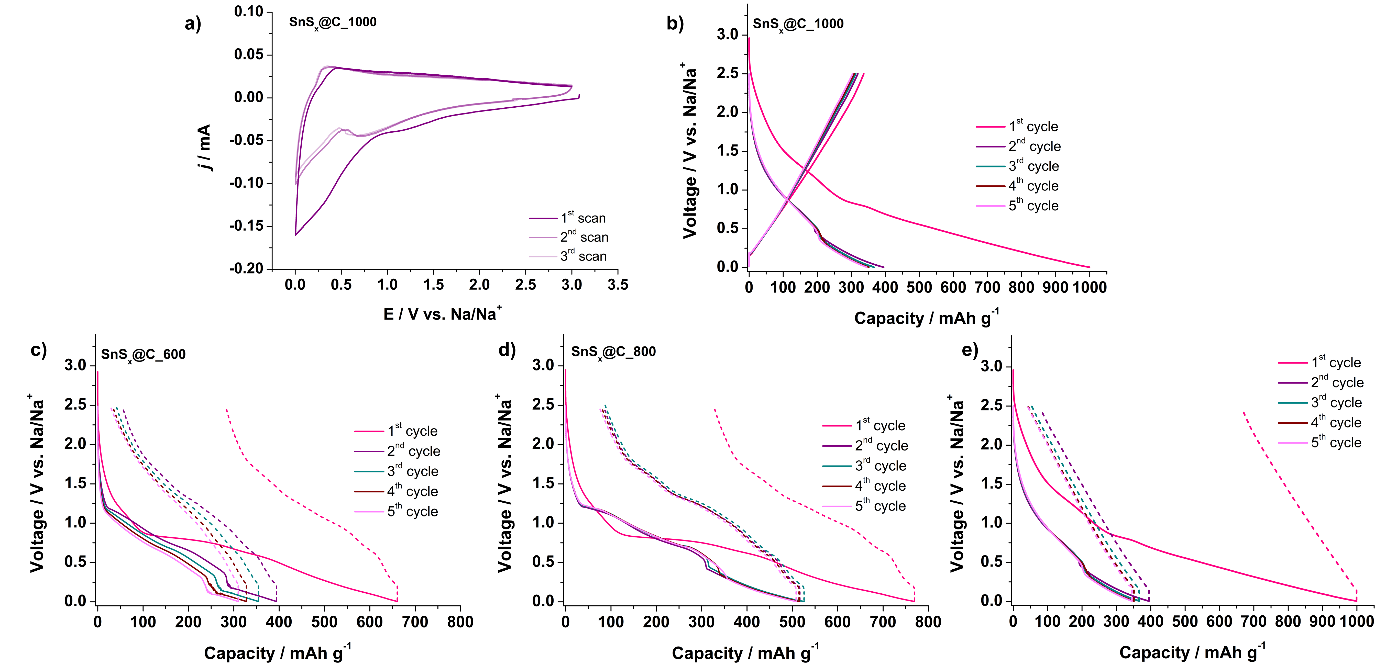
*

**Figure S6.** (a) Cyclic voltammetry curves recorded in 1 M NaPF_6_ in EC:DEC (30:70) + 5% FEC electrolyte (*v* = 100 µV s^-1^) for half-cells with the SnS*_x_*@C_1000 electrode material; (b) galvanostatic charge-discharge curves recorded in 1 M NaPF_6_ in EC:DEC (30:70) + 5% FEC at current density of *C*/10 for the SnS*_x_*@C_1000; galvanostatic charge-discharge curves exhibiting voltage hysteresis for (c) SnS*_x_*@C_600, (d) SnS*_x_*@C_800 and (e) SnS*_x_*@C_1000.

**Table S7.** Average charge and discharge voltages, with average voltage hysteresis calculated for SnS*_x_*@C_600, SnS*_x_*@C_800 and SnS*_x_*@C_1000 based on the charge/discharge curves in Figure S6.

|  | Cycle no. | $\bar{\boldsymbol{V}}$_sod_ $\boldsymbol{/V}$ | $\bar{\boldsymbol{V}}$_des_ $\boldsymbol{/V}$ | $\boldsymbol{\Delta}\bar{\boldsymbol{V}}$ $\boldsymbol{/V}$ |
| --- | --- | --- | --- | --- |
| SnS*_x_*@C_600 | 1 | 0.61 | 1.19 | 0.58 |
|  | 2 | 0.63 | 1.23 | 0.61 |
|  | 3 | 0.61 | 1.26 | 0.65 |
|  | 4 | 0.59 | 1.28 | 0.69 |
|  | 5 | 0.58 | 1.30 | 0.72 |
| SnS*_x_*@C_800 | 1 | 0.62 | 1.18 | 0.56 |
|  | 2 | 0.65 | 1.20 | 0.55 |
|  | 3 | 0.66 | 1.21 | 0.55 |
|  | 4 | 0.67 | 1.21 | 0.53 |
|  | 5 | 0.68 | 1.21 | 0.53 |
| SnS*_x_*@C_1000 | 1 | 0.69 | 1.24 | 0.55 |
|  | 2 | 0.60 | 1.25 | 0.65 |
|  | 3 | 0.64 | 1.25 | 0.61 |
|  | 4 | 0.66 | 1.25 | 0.60 |
|  | 5 | 0.66 | 1.25 | 0.59 |


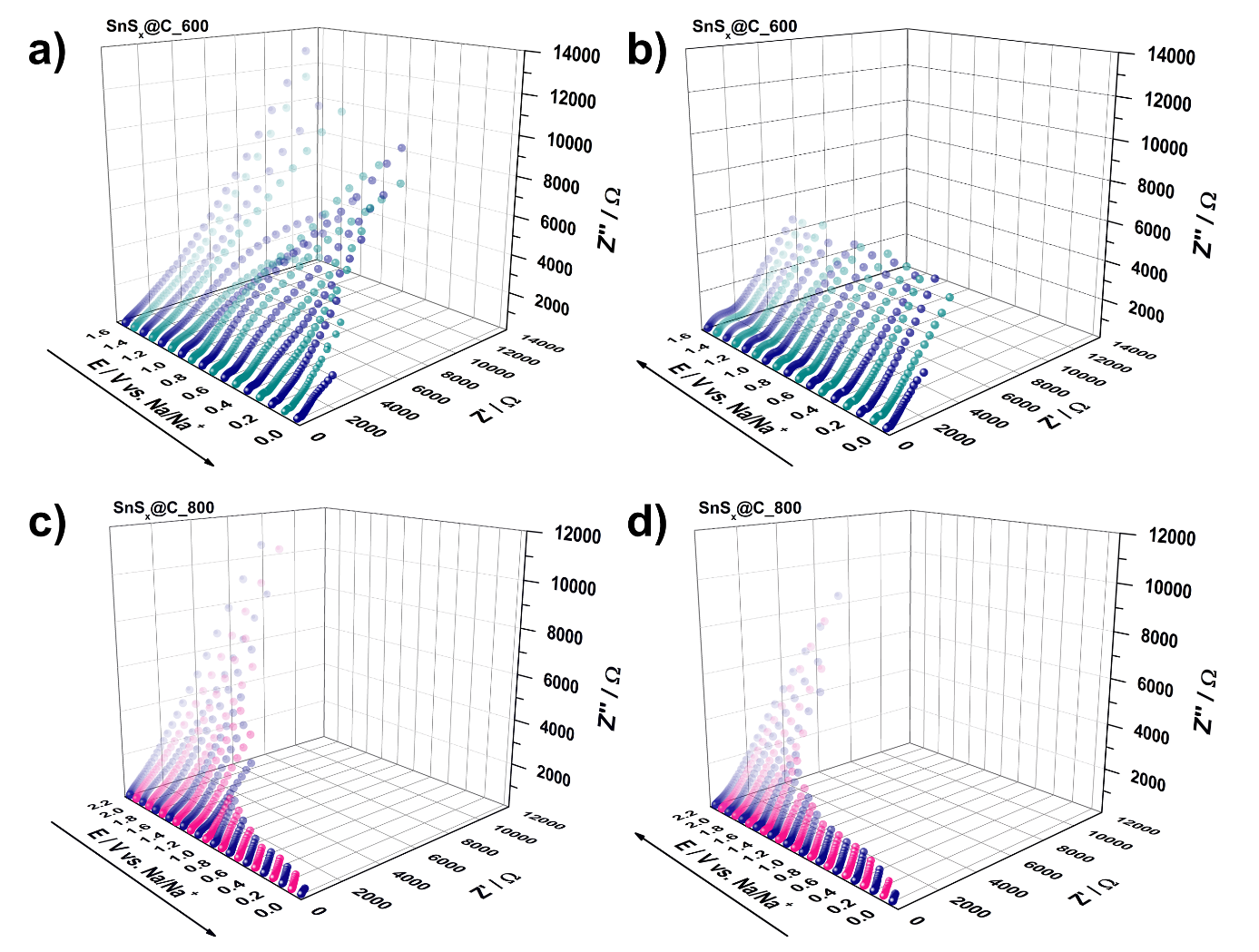


**Figure S7.** Staircase potentiostatic EIS results for (a-b) SnS*_x_*@C_600 and (c-d) SnS*_x_*@C_800 half-cells during sodiation (a, c) and desodiation (b, d), measured in the frequency range from 20 kHz to 1 mHz.


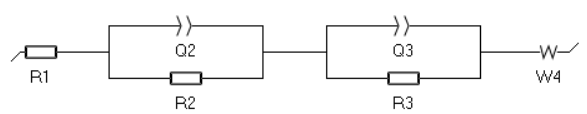


**Figure S8.** Electrical equivalent circuit used for Nyquist spectra fitting: R1 is the electrolyte resistance, R2/Q2 stands for the resistance (R_SEI_) and capacitance origination from SEI, R3/Q3 corresponds to the charge transfer (R_ct_) resistance and double layer capacitance, and W4 is a semi-infinite-length Warburg element that models Na^+^ diffusion.

**Table S8.** Impedance fitting data for SnS*_x_*@C_600 during desodiation cycle.

| ***SnS_x_@C_600*** | Desodiation | | | | |
| --- | --- | --- | --- | --- | --- |
| **E / V** | **R_SEI_ /** Ω | **R_ct_ /** Ω | **W_coeff_ /** Ω s^-1/2^ | **R^2^ (parametric)** | **R^2^ (amplitude)** |
| 0.1 | 99.58 | 106.28 | 123.35 | 1.51e-4 | 1.83e-05 |
| 0.2 | 42.82 | 397.32 | 297.20 | 6.47e-4 | 6.22e-05 |
| 0.4 | 25.79 | 411.43 | 448.99 | 7.27e-4 | 8.04e-05 |
| 0.6 | 59.81 | 434.52 | 722.60 | 1.30e-4 | 2.92e-05 |
| 0.8 | 82.67 | 499.89 | 873.78 | 2.16e-4 | 7.24e-05 |
| 1.0 | 55.35 | 543.08 | 942.39 | 8.35e-5 | 1.59e-05 |
| 1.2 | 52.37 | 852.99 | 1099.60 | 1.86e-6 | 3.31e-05 |
| 1.4 | 50.95 | 803.81 | 1265.00 | 5.43e-5 | 1.13e-05 |
| 1.6 | 52.50 | 1085.30 | 1384.5 | 5.03e-5 | 1.27e-05 |

**Table S9.** Impedance fitting data for SnS*_x_*@C_800 during desodiation cycle.

| ***SnS_x_@C_800*** | Desodiation | | | | |
| --- | --- | --- | --- | --- | --- |
| **E / V** | **R_SEI_ /** Ω | **R_ct_ /** Ω | **W_coeff_ /** Ω s^-1/2^ | **R^2^ (parametric)** | **R^2^ (amplitude)** |
| 0.1 | 12.61 | 20.77 | 12.17 | 2.49e-2 | 9.33e-06 |
| 0.2 | 17.91 | 19.96 | 17.33 | 2.50e-2 | 1.10e-05 |
| 0.4 | 25.38 | 23.22 | 27.36 | 3.13e-2 | 1.56e-05 |
| 0.6 | 24.21 | 25.73 | 29.52 | 2.40e-2 | 1.81e-05 |
| 0.8 | 30.76 | 34.86 | 32.29 | 2.42e-2 | 1.36e-05 |
| 1.0 | 33.38 | 29.90 | 40.14 | 2.07e-2 | 1.14e-05 |
| 1.2 | 40.92 | 56.83 | 59.89 | 1.59e-2 | 7.20e-06 |
| 1.4 | 52.30 | 61.01 | 82.67 | 1.34e-2 | 5.60e-06 |
| 1.6 | 80.86 | 76.68 | 119.32 | 1.38e-2 | 6.22e-06 |


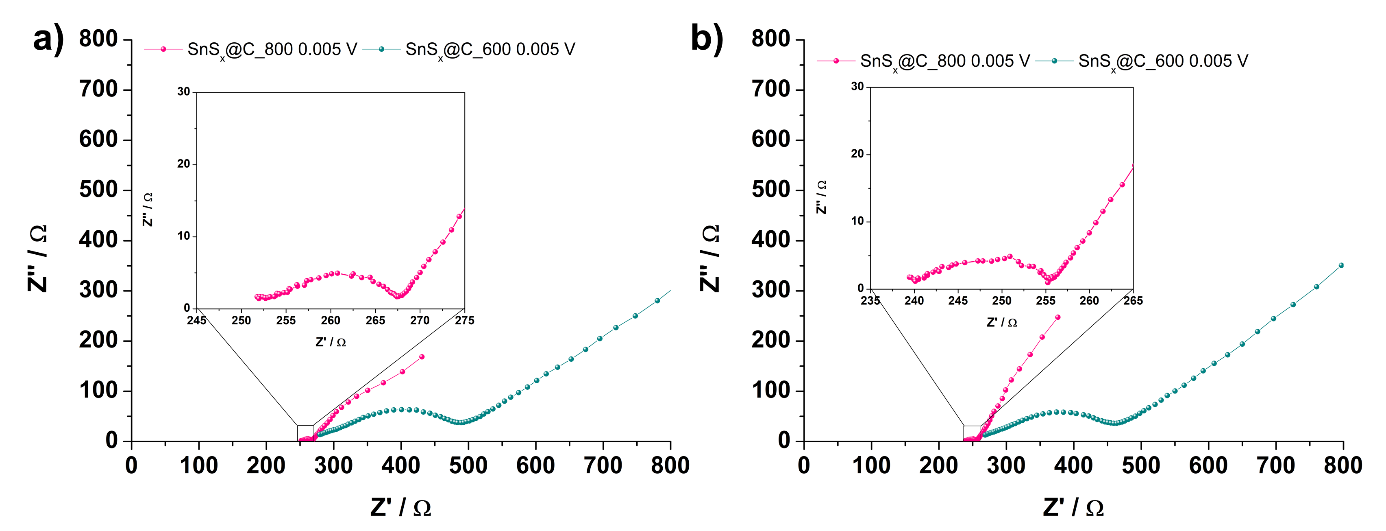


**Figure S9.** Nyquist plots for SnS*_x_*@C_600 and SnS*_x_*@C_800 recorded during a) sodiation and b) desodiation cycle at 0.005 V in the frequency range from 20 kHz to 1 mHz.

**Table S10.** Spectral parameters for the first-order Raman bands of SnS*_x_*@C_600 electrode material recorder during 2^nd^ charge/discharge test: band position (Stokes Raman Shift), half width at half maximum (HWHM), and **peak intensity ratios** relative to the G band.

| Band | Spectral parameter | Sodiation – 2^nd^ scan | | | Desodiation – 2^nd^ scan | | |
| --- | --- | --- | --- | --- | --- | --- | --- |
|  |  | 2.0 V | 0.7 V | 0.005 V | 0.005 V | 0.7 V | 2.0 V |
| G | Position (cm^-1^) | 1585.18 | 1585.38 | 1573.751 | 1573.751 | 1577.01 | 1579.43 |
|  | HWHM (cm^-1^) | 42.14 | 45.54 | 48.62 | 48.62 | 39.41 | 42.22 |
| D1 | Position (cm^-1^) | 1322.8 | 1329.03 | 1340.51 | 1340.51 | 1331.37 | 1328.27 |
|  | HWHM (cm^-1^) | 92.25 | 96.55 | 110.84 | 110.84 | 95.13 | 93.74 |
|  | **I_D1_/I_G_** | **1.74** | **1.79** | **1.44** | **1.26** | **1.92** | **1.71** |
| D3 | Position (cm^-1^) | 1500.12 | 1505.39 | 1505.52 | 1505.52 | 1505.67 | 1500.12 |
|  | HWHM (cm^-1^) | 91.46 | 79.71 | 55.52 | 55.52 | 77.95 | 81.04 |
|  | **I_D3_/I_G_** | **0.89** | **0.90** | **0.58** | **0.56** | **1.22** | **0.96** |
| D4 | Position (cm^-1^) | 1169.25 | 1166.23 | 1166.23 | 1166.23 | 1171.77 | 1169.25 |
|  | HWHM (cm^-1^) | 70.45 | 78.94 | 78.92 | 78.94 | 78.95 | 91.52 |
|  | **I_D4_/I_G_** | **0.49** | **0.47** | **0.22** | **0.04** | **0.45** | **0.49** |

**Table S11.** Spectral parameters for the first-order Raman bands of SnS*_x_*@C_800 electrode material recorder during 2^nd^ charge/discharge test: band position (Stokes Raman Shift), half width at half maximum (HWHM), and **peak intensity ratios** relative to the G band.

| Band | Spectral parameter | Sodiation – 2^nd^ scan | | | Desodiation – 2^nd^ scan | | |
| --- | --- | --- | --- | --- | --- | --- | --- |
|  |  | 2.0 V | 0.7 V | 0.005 V | 0.005 V | 0.7 V | 2.0 V |
| G | Position (cm^-1^) | 1592.25 | 1586.89 | 1569.47 | 1568.88 | 1577.35 | 1592.53 |
|  | HWHM (cm^-1^) | 45.13 | 38.13 | 33.61 | 32.63 | 41.77 | 39.23 |
| D1 | Position (cm^-1^) | 1323.49 | 1329.29 | 1339.53 | 1334.73 | 1339.49 | 1323.37 |
|  | HWHM (cm^-1^) | 88.72 | 88.32 | 88.56 | 86.94 | 89.34 | 85.15 |
|  | **I_D1_/I_G_** | **1.82** | **2.12** | **2.68** | **3.16** | **1.64** | **1.65** |
| D3 | Position (cm^-1^) | 1509.59 | 1509.47 | 1502.46 | 1502.01 | 1506.08 | 1501.86 |
|  | HWHM (cm^-1^) | 81.06 | 80.33 | 77.02 | 78.73 | 71.95 | 84.44 |
|  | **I_D3_/I_G_** | **0.83** | **0.84** | **1.96** | **2.33** | **0.85** | **0.83** |
| D4 | Position (cm^-1^) | 1176.15 | 1177.01 | 1186.01 | 1171.27 | 1189.84 | 1178.03 |
|  | HWHM (cm^-1^) | 74.77 | 91.28 | 108.39 | 81.20 | 82.15 | 84.73 |
|  | **I_D4_/I_G_** | **0.43** | **0.45** | **0.77** | **0.77** | **0.32** | **0.46** |


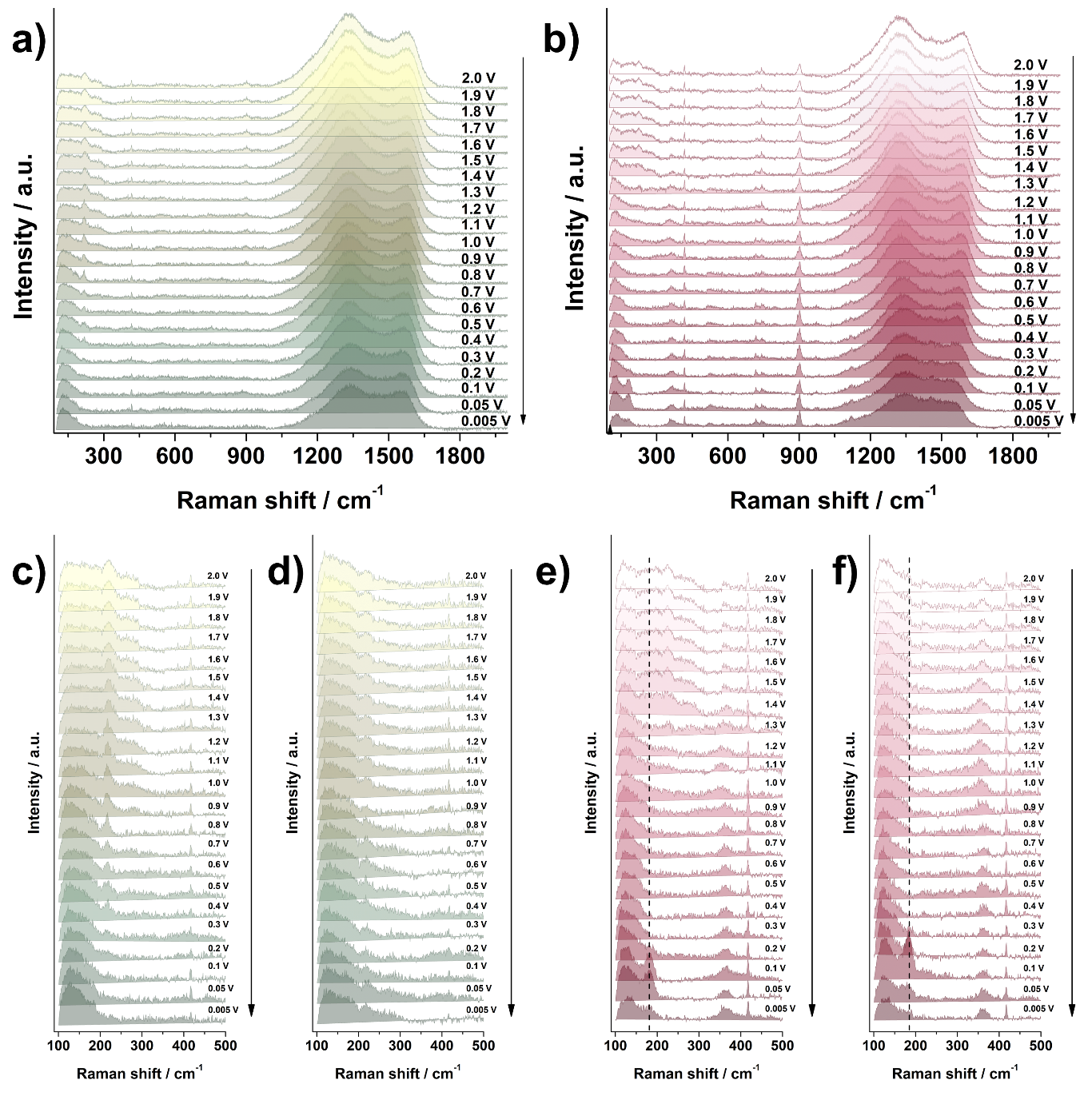


**Figure S10.** Raman spectra in a full range recorded during sodiation for (a) SnS*_x_*@C_600 (2^nd^ cycle) and (b) SnS*_x_*@C_800 (2^nd^ cycle); 100 – 800 cm^-1^ range for (c) SnS*_x_*@C_600 (2^nd^ cycle), (d) SnS*_x_*@C_600 (20^th^ cycle), (e) SnS*_x_*@C_800 (2^nd^ cycle), (f) SnS*_x_*@C_800 (20^th^ cycle).

**Table S12.** Spectral parameters for the first-order Raman bands of SnS*_x_*@C_600 electrode material recorder during 20^th^ charge/discharge test: band position (Stokes Raman Shift), half width at half maximum (HWHM), and **peak intensity ratios** relative to the G band.

| Band | Spectral parameter | Sodiation – 20^th^ scan | | | Desodiation – 20^th^ scan | | |
| --- | --- | --- | --- | --- | --- | --- | --- |
|  |  | 2.0 V | 0.7 V | 0.005 V | 0.005 V | 0.7 V | 2.0 V |
| G | Position (cm^-1^) | 1587.09 | 1587.27 | 1587.68 | 1588.10 | 1587.99 | 1587.74 |
|  | HWHM (cm^-1^) | 38.79 | 45.43 | 42.38 | 39.46 | 40.85 | 39.92 |
| D1 | Position (cm^-1^) | 1325.48 | 1328.46 | 1327.75 | 1324.32 | 1328.10 | 1326.16 |
|  | HWHM (cm^-1^) | 88.97 | 90.35 | 88.30 | 93.06 | 88.17 | 86.48 |
|  | **I_D1_/I_G_** | **1.71** | **1.63** | **1.70** | **1.89** | **1.75** | **1.68** |
| D3 | Position (cm^-1^) | 1500.12 | 1500.51 | 1500.48 | 1503.51 | 1501.03 | 1500.03 |
|  | HWHM (cm^-1^) | 85.71 | 81.98 | 82.40 | 85.90 | 87.36 | 87.96 |
|  | **I_D3_/I_G_** | **0.96** | **0.89** | **0.93** | **1.03** | **1.03** | **0.99** |
| D4 | Position (cm^-1^) | 1176.65 | 1176.65 | 1174.33 | 1162.344 | 1179.42 | 1181.14 |
|  | HWHM (cm^-1^) | 71.46 | 71.43 | 71.40 | 71.46 | 78.09 | 75.53 |
|  | **I_D4_/I_G_** | **0.47** | **0.49** | **0.53** | **0.59** | **0.53** | **0.48** |

**Table S13.** Spectral parameters for the first-order Raman bands of SnS*_x_*@C_800 electrode material recorder during 20^th^ charge/discharge test: band position (Stokes Raman Shift), half width at half maximum (HWHM), and **peak intensity ratios** relative to the G band.

| Band | Spectral parameter | Sodiation – 20^th^ scan | | | Desodiation – 20^th^ scan | | |
| --- | --- | --- | --- | --- | --- | --- | --- |
|  |  | 2.0 V | 0.7 V | 0.005 V | 0.005 V | 0.7 V | 2.0 V |
| G | Position (cm^-1^) | 1591.15 | 1587.34 | 1564.32 | 1565.09 | 1577.46 | 1592.04 |
|  | HWHM (cm^-1^) | 34.32 | 38.65 | 35.75 | 35.74 | 37.05 | 37.68 |
| D1 | Position (cm^-1^) | 1324.41 | 1324.41 | 1338.72 | 1339.78 | 1339.83 | 1324.81 |
|  | HWHM (cm^-1^) | 83.66 | 86.43 | 89.17 | 90.86 | 89.12 | 83.98 |
|  | **I_D1_/I_G_** | **1.80** | **1.77** | **2.75** | **2.78** | **1.73** | **1.58** |
| D3 | Position (cm^-1^) | 1508.40 | 1507.57 | 1502.30 | 1505.69 | 1508.25 | 1501.67 |
|  | HWHM (cm^-1^) | 89.14 | 82.40 | 73.63 | 73.62 | 71.26 | 79.57 |
|  | **I_D3_/I_G_** | **0.99** | **0.91** | **1.98** | **2.00** | **1.02** | **0.81** |
| D4 | Position (cm^-1^) | 1182.23 | 1171.21 | 1171.37 | 1178.99 | 1180.06 | 1178.97 |
|  | HWHM (cm^-1^) | 88.06 | 83.83 | 80.14 | 80.06 | 78.64 | 88.55 |
|  | **I_D4_/I_G_** | **0.47** | **0.42** | **0.54** | **0.36** | **0.31** | **0.42** |


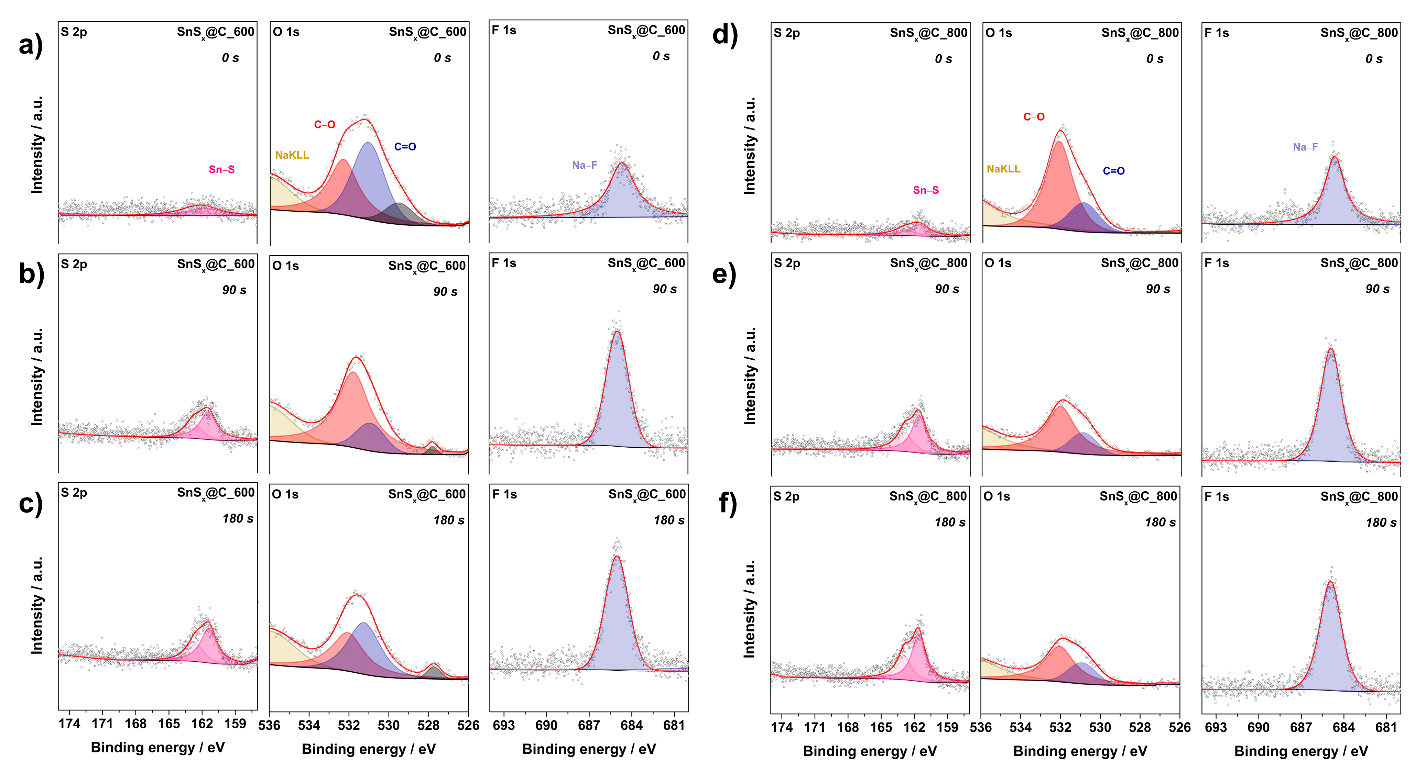


**Figure S11.** XPS depth-profiling results for (a-c) SnS*_x_*@C_600 and (d-f) SnS*_x_*@C_800 at (a, d) 0 s, (b, e) 90 s, (c, f) 180 s of Ar^+^ sputtering.
